# TOR–NOT4 signalling couples translation to co-translational quality control in plants

**DOI:** 10.1038/s41467-026-77200-0

**Published:** 2026-08-24

**Authors:** Maximillian A. Schwarze, Rory Osborne, Margot Raffeiner, Manuel González-Fuente, Anastasia Kanali, Mark Bailey, Ross D. Etherington, Lina Zhou, Suayib Üstün, Daniel J. Gibbs

**Affiliations:** 1 School of Biosciences, University of Birmingham, Birmingham, B15 2TT UK; 2 Faculty of Biology and Biotechnology, Ruhr-University Bochum, Bochum, Germany

**Keywords:** Proteolysis in plants, Plant signalling, Plant molecular biology, Plant physiology

## Abstract

Protein synthesis must be tightly coordinated with quality control to prevent proteotoxic stress, yet the mechanisms underlying co-translational surveillance in plants, and how these are aligned with translational output, remain poorly understood. Here, we identify three NOT4-like E3 ubiquitin ligases in *Arabidopsis thaliana* as regulators of co-translational protein quality control and uncover a functional link between NOT4 and TARGET OF RAPAMYCIN (TOR) signalling that coordinates quality-control capacity with translational output. Loss of NOT4 function increases basal TOR activity and global translation rates, resulting in the accumulation of polyubiquitylated proteins and heightened sensitivity to proteasome inhibition, TOR inhibition, and protein misfolding stress. Consistent with prior evidence that NOT4 proteins are TOR-regulated phosphotargets, *not4* mutants also phenocopy TOR-inhibited wild-type plants for a subset of transcriptional and growth-related processes. Furthermore, elevated translation in NOT4-deficient plants enhances resistance to *Pseudomonas syringae* pv. *tomato*. Collectively, our findings reveal a functional coupling between TOR signalling and NOT4 activity that may scale quality control with translational demand to safeguard proteome homoeostasis across eukaryotes.

## Abstract (web summary)

Here, Schwarze et al. reveal how plant cells match protein quality control to changing rates of protein production through a functional link between TOR kinase signalling and NOT4 E3 ubiquitin ligases

## Introduction

Proteins are essential components of cells, and the proteome - i.e., the complete cohort of proteins and their modification status - must be effectively regulated to maintain cellular integrity. This is achieved through the dynamic balance of protein production, folding, modification, and destruction - processes that collectively coordinate protein homoeostasis (proteostasis)^1^. A critical contributory step to proteome function is protein synthesis, where mRNAs are decoded and translated into polypeptides by the ribosome. However, problems can arise during this process, leading to the production of misfolded or otherwise aberrant proteins that negatively impact cellular function. For example, mRNA errors can trigger No-Go (NGD), Non-Stop (NSD) and Nonsense-Mediated (NMD) decay, causing ribosome stalling, mRNA read-through, and premature protein truncation^2–6^. In parallel, nascent proteins that stem from these defective mRNAs or that emerge due to incorrect codon assignment can either accumulate on stalled polysomes or be released in a non-optimally folded state, potentially leading to proteotoxicity. To mitigate the former, erroneous proteins are co-translationally polyubiquitylated and degraded by the 26S proteasome^7,8^. The co-ordinated depletion of defective mRNAs and proteins through these complementary pathways is required to ensure that only correctly formed proteins make it to maturity in the cell^3^.

Several Ribosome-associated Quality Control (RQC) E3 ubiquitin ligases have been described in yeast and mammals^7,9^, including LTN1/Listerin^10^, HEL2/RQT1^11,12^ and NOT4^13^. While LTN1 and HEL2 directly bind to the 60S and 40S ribosomal subunits, respectively^14,15^, NOT4 associates with polysomes as part of the multi-subunit CCR4-NOT complex that is also critical for regulating 3’−5’ mRNA decay, and which has broader roles in genome control and translational repression^16–24^. Loss of LTN1 and/or HEL2 function reduces the levels of ubiquitylated nascent ribosome-associated polypeptides, while loss of NOT4 causes an overaccumulation of polyubiquitylated and aggregated proteins due to problems in mRNA quality control (QC), causing increased error rates and triggering ubiquitylation by downstream E3 ligases. In contrast to yeast and mammals, knowledge of RQC pathways in plants is lagging^25^. Recently, LTN1- and HEL2-like proteins have been described in rice^26–28^, and shown to have broadly conserved functions, though their activity has to date only been investigated within the context of Thermo-Sensitive Genic Male Sterility (TGMS), and their wider roles across plant species, developmental stages, and stress conditions relevant to growth and immunity remain to be fully defined. Beyond the identity of these factors, it remains unclear how co-translational surveillance machineries are dynamically scaled to match changes in translational output under optimal and stress conditions.

An important regulator of protein translation rates in eukaryotic organisms is the TARGET OF RAPAMYCIN (TOR) kinase, which in association with RAPTOR and LST8 forms the conserved TORC1 complex^29,30^. This complex is critical for aligning protein synthesis and growth rates with nutrient and energy availability and is also responsive to phytohormone and stress signals^31–33^. TORC1 function is transduced through the phosphorylation of diverse substrates, including cell cycle regulators, several eukaryotic initiation factors, and the S6K kinase that targets Ribosomal protein S6 (RPS6) to increase translational activity and enhance the expression of ribosomal proteins^34,35^. Given its central role in driving translational output, it is plausible that TOR activity must be coordinated with protein QC systems to prevent proteotoxic stress; however, a functional link between TOR signalling and co-translational QC has not yet been established. Interestingly, a recent large-scale screen for TORC1 interactors and phospho-targets in *Arabidopsis thaliana* (hereafter Arabidopsis) identified a broad range of direct and indirect substrates, including putative homologues of yeast and mammalian NOT4 E3 ligases, and many that have not been characterised before^36,37^, raising the possibility that TOR may directly engage components of co-translational QC pathways.

Here, we identify three NOT4-like E3 ubiquitin ligases in Arabidopsis and establish their importance in co-translational protein QC. We show that NOT4 function influences translational homoeostasis and proteotoxic stress in plants and uncover a functional connection between TOR signalling and NOT4 activity. Building on prior evidence that NOT4 proteins are TOR-regulated phosphotargets, our findings support a model in which TOR signalling coordinates co-translational QC through NOT4. Given the evolutionary conservation of both TOR and NOT4 proteins, this TOR–NOT4 signalling module may operate more broadly across eukaryotes to align translational throughput with protein QC demand during growth, development and in response to environmental challenge.

## Results

### Identification and functional analysis of Arabidopsis NOT4-like proteins

**Fig. 1:**
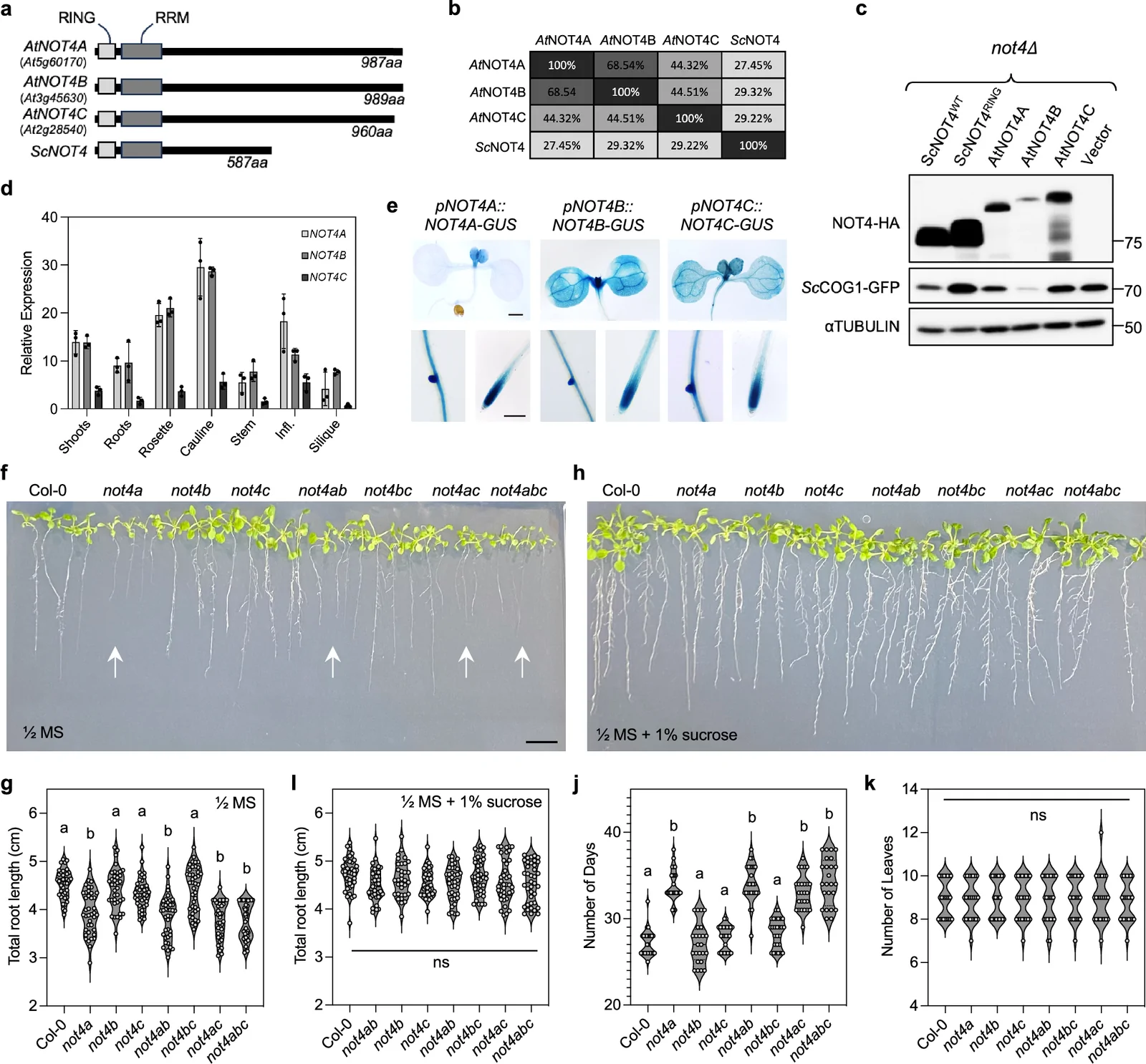
Identification and phenotypic characterization of *Arabidopsis* NOT4-like proteins. **a** Schematic showing overall domain structure and amino acid (aa) length of Arabidopsis (*At*) and yeast (*Sc*) NOT4-like proteins. **b** Sequence identity matrix between NOT4 orthologs. **c** Western blot of *At* and *Sc* NOT4-HA variants co-expressed with *Sc*COG1-GFP in the yeast *not4Δ* mutant. Steady-state levels of *Sc*COG1-GFP, a known *Sc*NOT4 degradation target, are depleted in the presence of *Sc*NOT4-HA and *At*NOT4A-B-HA, relative to catalytically inactive *Sc*NOT4^RING^-HA and the vector-only control. Tubulin and Coomassie Brilliant Blue (CBB) are shown as loading controls. HA-tagged *At*NOT4 proteins displayed apparent molecular weights slightly above those predicted from sequence. **d** qRT-PCR of *AtNOT4A-C* in different *Arabidopsis* tissues (infl. = inflorescence). n = 3 biological replicates. **e** Histochemical staining of 7-day-old *pNOT4::NOT4A-C-*GUS seedlings. Scale bar = 500 μm. **f**, **g** Representative image and quantification of primary root (PR) lengths of 14-day-old seedlings grown on ½ MS medium. White arrows highlight genotypes with significantly shorter PRs. Scale bar = 1 cm. **h**, **i** Same as in (**f**) and (**g**), but with 1% sucrose supplemented ½ MS. **j** Number of days to flowering for denoted genotypes. **k** Number of rosette leaves at flowering for denoted genotypes (ns = not significant). Statistically significant differences in (**g**), (**i**), (**j**) and (**k**) were calculated by one-way ANOVA followed by Tukey’s test, presented in compact letter display (CLD) format. All error bars in this figure represent ± SEM.

To investigate how co-translational QC is coordinated with protein synthesis in plants, we sought to identify conserved components that could link translational activity to proteostasis. In yeast and animals, NOT4 E3 ubiquitin ligases are established components of co-translational QC pathways. However, whether analogous mechanisms operate in plants remains unclear. Using reciprocal BLASTs with full-length yeast (*Sc*) protein sequences, we identified three NOT4-like protein sequences in the Arabidopsis genome (*At*NOT4A, *At5g60170*; *At*NOT4B, *At3g45630* and *At*NOT4C, *At2g28540*). All three have a similar N-terminal domain structure to *Sc*NOT4, including the characteristic RING domain and an RNA recognition motif (RRM) pairing, followed by a significantly longer C-terminal region (Fig. 1a). Full-length sequence identity between *At*NOT4s and *Sc*NOT4 was ~30%, with higher conservation ( ~ 45–70%) within the functionally conserved domains (Fig. 1b and Supplementary Fig. 1a, b). To test for potential functional homology, we co-expressed C-terminally HA-tagged variants of yeast and Arabidopsis NOT4s with the known *Sc*NOT4 degradation target COG1-GFP^38^ in a yeast *not4*Δ mutant. Whereas mutant *Sc*NOT4^RING^-HA (which lacks RING domain activity)^39^ had no effect on the steady-state levels of COG1-GFP, WT *Sc*NOT4-HA, *At*NOT4A-HA, and particularly *At*NOT4B-HA, led to COG1-GFP depletion relative to the vector control (Fig. 1c). Together, these findings indicate partial functional conservation of NOT4 activity between yeast and Arabidopsis, consistent with a conserved role in co-translational QC.

RT-qPCR analysis of endogenous transcripts and histochemical staining of seedlings expressing *pNOT4::NOT4A-C-GUS* translational fusions revealed that *AtNOT4* genes are broadly expressed across tissues throughout the Arabidopsis lifecycle, with some variation between isoforms (Fig. 1d, e). We identified single homozygous T-DNA insertion lines for all three genes, and generated double and triple combination mutants, confirming homozygous T-DNA insertion and full-length mRNA knockout by PCR, RT-qPCR and RNA-sequencing (RNA-seq; see later) (Supplementary Fig. 1c–e). Using primer sets both upstream and downstream of the T-DNA insertion sites, we observed strong positive feedback on expression of all three genes when at least one was disrupted, consistent with functional redundancy and a constitutive requirement for normal cellular function (Supplementary Fig. 1d).

The *not4a* single mutant and associated combination mutants (*not4ab, not4ac* and *not4abc*) grown as seedlings on ½ MS media had significantly reduced primary root lengths and slower overall growth compared to Col-0 (Fig. 1f, g). To further validate that the observed developmental phenotype results from disruption of *NOT4A*, we introduced a *35S::NOT4A-eGFP* transgene into the *not4a* background and observed substantial rescue of the reduced primary root phenotype (Supplementary Fig. 2a, b). The slow growth phenotypes persisted through the rosette stage under soil-grown conditions and resulted in delayed flowering under long-day photoperiods (16:8 h Light:Dark) (Fig. 1j and Supplementary Fig. 2b). In contrast, developmental flowering time was unaffected, with all lines producing a similar number of rosette leaves at bolting (Fig. 1k), suggesting that the phenotype does not arise from altered developmental timing, but may instead reflect altered energy supply, acquisition or mobilisation. Supporting this, supplementation of ½ MS media with 1% sucrose restored primary root growth to near wild-type levels (Fig. 1h, i). However, *not4abc* seedlings displayed normal recovery following extended dark treatment, comparable to WT but reduced compared to the carbon-starvation–tolerant *prt6* mutant^40^ (Supplementary Fig. 2c). These results suggest that loss of NOT4 function does not directly impair energy availability or mobilisation, but instead is consistent with altered signalling and/or proteostasis.

### Elevated translation rates in *not4* mutants

**Fig. 2:**
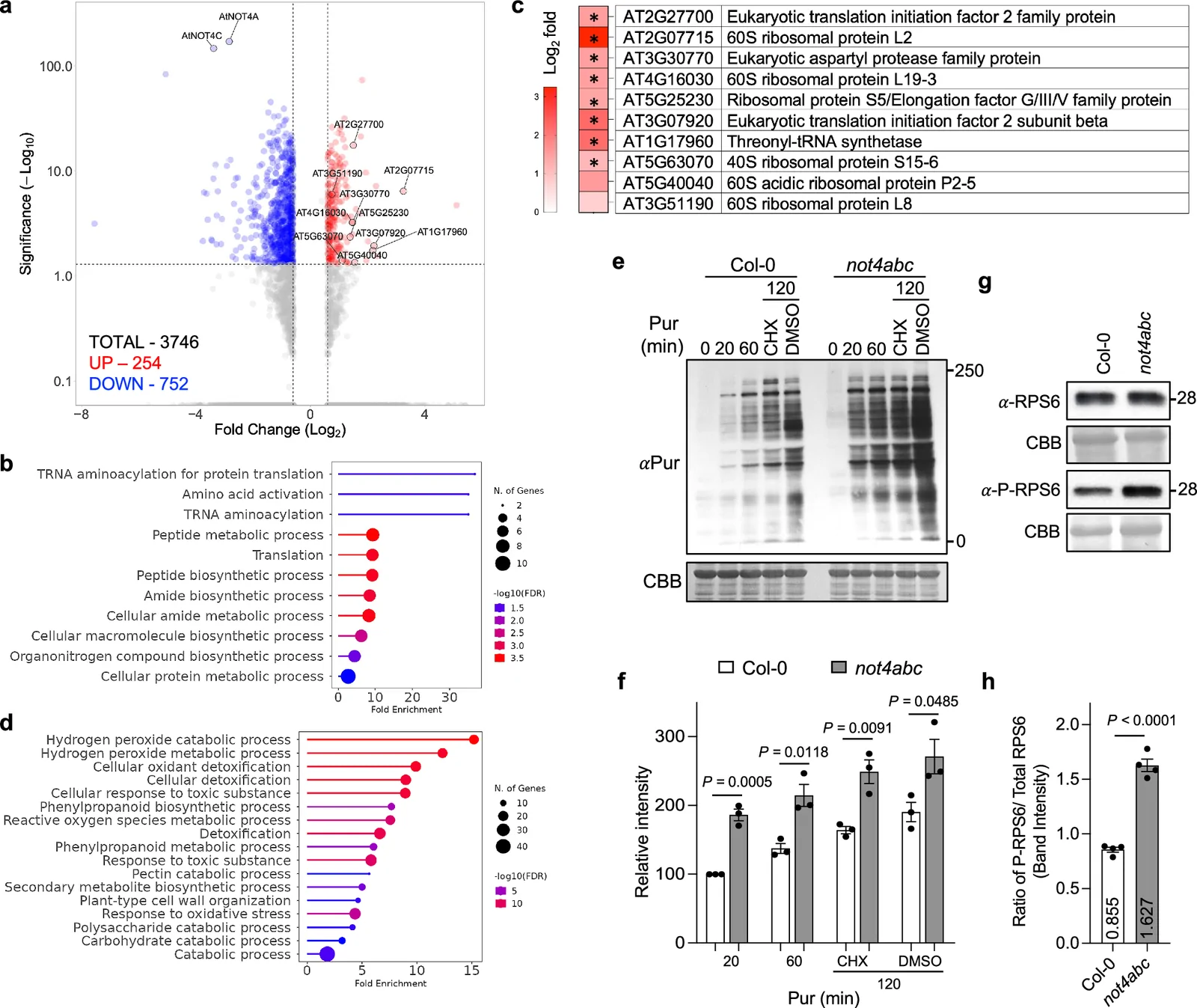
Elevated translation rates in *not4* mutants. **a** Volcano plot of differentially expressed genes in 12-day-old *not4ab*c vs WT seedlings grown on ½ MS medium (3 biological replicates; *n* = 3). The x-axis shows Log_2_ fold change; the y-axis shows significance (-Log10 padj value). Significantly up- and down-regulated (1.5FC, padj <0.05) genes are shown in red and blue, respectively. Labels denote translation-associated upregulated genes (see also c). Differential expression was determined using DESeq2, with P values adjusted for multiple testing using the Benjamini–Hochberg method. **b** Lollipop plot showing significant GO terms in the top 10% of *not4abc* up DEGs, ranked by fold enrichment. (**c**) Lollipop plot showing significant GO terms in the top 10% down DEGs, ranked by fold enrichment. In (**b**) and (**c**), the X-axis shows fold enrichment, circle size shows the number of genes, and colour denotes significance (-Log_10_ FDR). **d** Heat map showing Log_2_ fold change (padj <0.05) in *not4abc* of genes linked to protein translation, also labelled on the volcano plot in (**a**). Asterisks denote genes that are in the top 10% most strongly up DEGs. **e** anti-Puromycin Western blot of total protein extracted from 12-day-old seedlings following treatment with puromycin for the times indicated. Cycloheximide (CHX) or DMSO were added at 60 minutes to inhibit, or not, translation, and seedlings were incubated for a further 60 minutes. Loading control = Coomassie Brilliant Blue (CBB). **f** Quantification of puromycin incorporation in (**e**), based on three biological reps (*n* = 3; see also Fig. S3g). Statistically significant differences were calculated by unpaired two-tailed Student’s *t*-test with exact P values shown. **g** Steady state levels of total (RPS6) and phosphorylated (P-RPS6) Ribosomal Protein S6A (RPS6A) in 12-day-old Col-0 and *not4abc* seedlings. CBB-loading control. Approximate molecular weight in kDa is shown. **h** Average quantified ratio of P-RPS6 to RPS6 calculated from 4 independent biological reps (*n* = 4). Values are shown within each bar. Statistically significant differences were calculated by unpaired two-tailed Student’s *t*-test with P value shown. All error bars in this figure represent ± SEM.

To investigate the molecular consequence of NOT4 disruption under normal growth conditions, we performed RNA-seq on 12-day-old *not4abc* vs WT seedlings, which identified 254 up and 752 down differentially expressed genes ( > 1.5 FC, padj <0.05) (Fig. 2a and Supplementary Data 1). A gene ontology (GO) analysis of the most highly upregulated DEGs revealed a significant overrepresentation of terms linked to translation and peptide biosynthesis (Fig. 2b). Prominent amongst these genes were several eukaryotic translation initiation complex subunits and elongation factors, as well as 40S and 60S ribosomal subunits and aminoacyl tRNA synthetases (Fig. 2a, c). A closer examination of genes falling just below the adjusted significance threshold revealed many additional upregulated genes (>1.5 FC) associated with protein synthesis and ribosome biogenesis (Supplementary Fig. 3a, b). *not4abc* downregulated genes were associated with GO terms linked to cell wall organisation and biogenesis (e.g., extensins, expansins and xyloglucans), Casparian strip formation (e.g., CASPs and associated peroxidases), as well as ROS metabolism and detoxification (Fig. 2d and Supplementary Fig. 3c–e). Also prominent in the downregulated gene set were transcripts associated with amino acid, hormone, and nutrient transport (Supplementary Fig. 3f).

The upregulation of genes linked to protein synthesis led us to hypothesise that loss of NOT4 function might disrupt the balance between translational output and protein QC, resulting in elevated translation rates. To investigate this, we monitored protein synthesis in 12-day-old seedlings using incorporation of the tyrosyl-tRNA structural analogue puromycin into nascent polypeptide chains over time^41^. Following incubation with puromycin, anti-puromycin Western blotting was used to detect and quantify levels of nascent puromicylated proteins. This revealed significantly faster synthesis rates in *not4abc* that were evident within 20 minutes ( ~ 2-fold) and continued over the 2 hour timeframe tested (Fig. 2e,f and Supplementary Fig. 3g). Following 60 minutes of puromycin treatment, addition of cycloheximide (CHX) suppressed further puromycin incorporation in both genotypes, whereas DMSO-treated seedlings continued to accumulate puromicylated proteins over the subsequent 60 minutes. Together, these observations confirm that the detected signal reflects active translation (Fig. 2e). We also monitored TOR kinase activity by determining the ratio of phosphorylated to non-phosphorylated RPS6A, a ribosomal subunit whose phosphorylation status is frequently used as a proxy for global translational activity^34^. Corroborating the elevated translation rates observed, *not4abc* had an almost 2-fold increase in basal TOR activity relative to WT, independent of any changes in the expression levels of TORC1 components (Fig. 2g, h and Supplementary Fig. 5g). Thus, our RNA-seq and biochemical analyses reveal that loss of NOT4 function leads to an upregulation of the protein translation machinery and increased translation activity.

### Loss of NOT4 function leads to proteotoxic stress

**Fig. 3:**
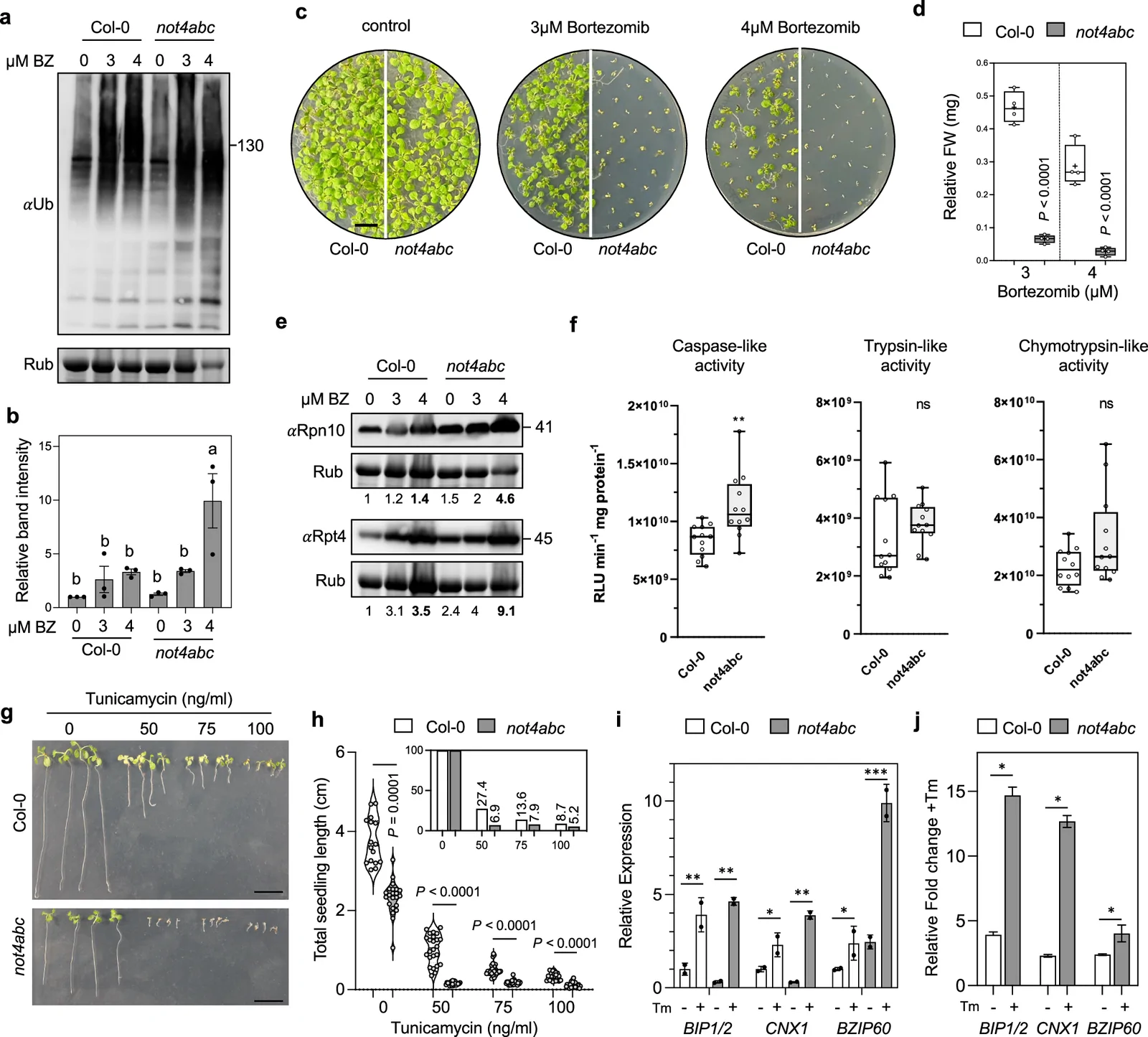
Loss of NOT4 function leads to proteotoxic stress. **a** anti-polyubiquitin (αUb) Western blot of total protein extracts from 14-day-old seedlings of Col-0 and *not4abc* treated +/− 3 μM or 4 μM Bortezomib (BTZ). **b** Quantification of total polyubiquitylated protein in Col-0 and *not4abc* treated as in (**a**), averaged across 3 biological replicates (*n* = 3). Significant differences were calculated by one-way ANOVA followed by Tukey’s test, presented in compact letter display (CLD) format. (**c**) Representative images of 14-day-old seedlings treated +/− 3 μM or 4 μM Bortezomib (BTZ). **d** Relative fresh weight of 14-day-old Col-0 and *not4abc* seedlings treated +/− 3 μM or 4 μM BTZ. Each replicate is the mean pool of 6 seedlings taken from 2 separate plates. Statistically significant differences were calculated by unpaired two-tailed Student’s t-test with P value shown. Scale bar = 1 cm. **e** Western blot of steady state levels of Rpn10 and Rpt4 in 14-day-old seedlings of Col-0 and *not4abc* treated +/− 3 μM or 4 μM BTZ. Rub = Rubisco loading control. Quantified values beneath the blot represent RPN10 and RPT4 intensities normalized to the corresponding Rubisco loading signal. **f** 26S proteasome protease activities. Protease activities are indicated in relative luminescence units (RLUs) min^−1^. Data are pooled from 3 independent experiments, each with 4 biological reps (n = 12). Statistically significant differences were calculated using an unpaired two-tailed Student’s *t*-test: **p < 0.01. **g** Representative image of 12-day-old seedlings grown on ½ MS medium supplemented with increasing concentrations of Tunicamycin (Tm). Scale bar = 1 cm. **h** Violin plot quantifying total primary root length of seedlings grown as in (**g**). Statistically significant differences were calculated by unpaired two-tailed Student’s t-test with *P* values shown. Inset graph shows primary root length expressed as a percentage of the corresponding untreated control for each genotype (untreated = 100). **i** Relative expression of UPR marker genes *BIP1/2*, *CNX1* and *BZIP60* in 12-day-old Col-0 and *not4abc* seedlings grown on ½ MS+/− 50 ng/ml Tm. Results are the average of 2 biological replicates (*n* = 2), normalized to 2 housekeeping genes, with relative expression calculated against Col-0 (-Tm) for each gene tested. Statistically significant differences were calculated by using a paired two-tailed Wilcoxon signed-rank test: *p < 0.05, **p < 0.01, ***p < 0.001. **j** Relative fold change upon Tm treatment for Col-0 and *not4abc* in (**i**). *n* = 2 biological replicates. Statistically significant differences were calculated using a two-tailed Mann-Whitney U-test: *p < 0.05. All error bars in this figure represent ± SEM. For box plots in (**d**) and (**f**), boxes extend from the 25th to 75th percentiles, the centre line indicates the median, and whiskers extend from the minimum to maximum values.

Given the requirement for co-translational QC to be coordinated with translational output, we reasoned that elevated translation in *not4abc* would exceed QC capacity, leading to proteotoxic stress. We therefore assessed whether an increase in protein synthesis rates in the absence of NOT4s leads to the accumulation of aberrant translational products, which would need to be ubiquitylated and cleared by the 26S proteasome. Accordingly, we observed hyperaccumulation of total polyubiquitylated proteins (Fig. 3a, b) and strong inhibition of seedling growth (Fig. 3c, d) in *not4abc* vs WT when treated with increasing concentrations of the 26S proteasome inhibitor bortezomib (BZ). By contrast, the individual *not4* single mutants did not exhibit enhanced sensitivity to bortezomib treatment relative to Col-0 (Supplementary Fig. 2e), indicating that the proteotoxic stress phenotype only emerges following combined loss of NOT4 function. Consistent with this heightened sensitivity, elevated levels of the representative proteasome subunits RPN10 and RPT4 were observed in *not4abc* relative to Col-0 following bortezomib treatment, based on quantification normalized to the corresponding Rubisco loading signal (Fig. 3e). Furthermore, an assessment of total 26S proteasome activity levels revealed increased rates of caspase-like proteasome proteolytic activity in *not4abc*, though no significant differences in trypsin- or chymotrypsin-like activity were observed (Fig. 3f). Together, these data reveal that loss of NOT4 function leads to a heightened state of proteotoxic stress.

We further reasoned that a build-up of malformed proteins in *not4abc* might also overwhelm the plant’s post-translational QC systems, rendering *not4abc* mutants more sensitive to additional protein misfolding stressors. Supporting this hypothesis, and in keeping with the general proteotoxicity phenotypes observed, we found that *not4abc* seedlings were hypersensitive to the protein misfolding elicitor tunicamycin relative to WT (Fig. 3g, h). Basal expression of the canonical UPR marker genes *BIP1/2*, *CNX1* and *BZIP60* was not elevated in *not4abc*, with *BIP1/2* and *CNX1* instead showing modestly reduced expression under untreated conditions, consistent with the RNA-seq data (Supplementary Fig. 3h). However, all three marker genes were hyperinduced relative to WT in response to tunicamycin treatment (Fig. 3i, j), indicating that *not4abc* does not constitutively activate the canonical UPR but is hypersensitive to additional ER protein-folding stress. Together, these assays indicate that elevated translation rates following loss of NOT4 function place increased burden on the ubiquitin-proteasome system, highlighting a key role for NOT4s in co-translational QC and proteostasis.

### NOT4 modulates TOR-dependent signalling outputs

**Fig. 4:**
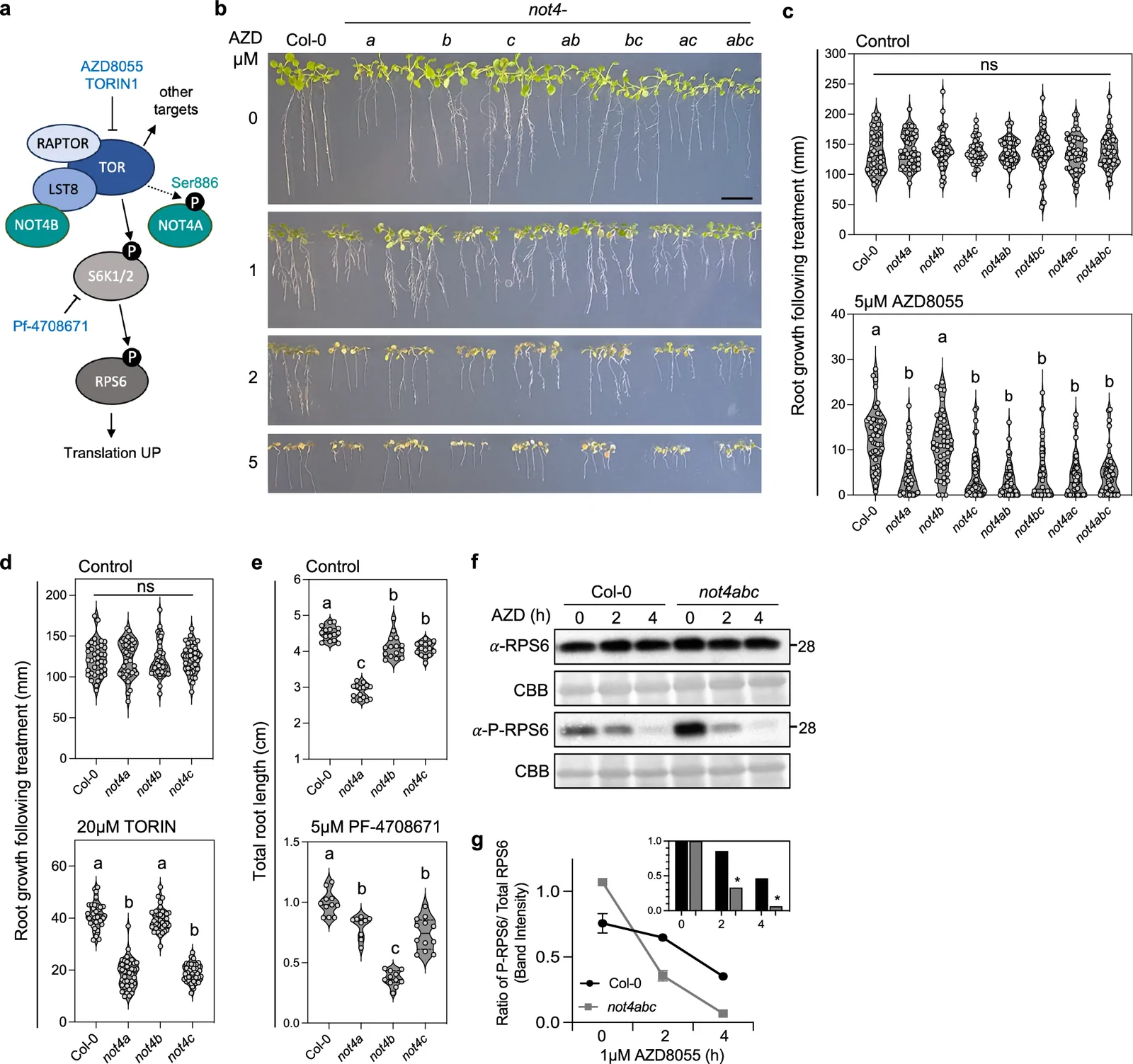
Loss of NOT4 activity leads to TOR signalling defects. **a** Schematic of the RPS6 branch of TOR signalling, showing the TORC1 complex (TOR, RAPTOR and LST8), which when activated initiates a phosphorylation (P) cascade through S6K1/2 to RPS6 to increase translation. NOT4A was shown to be phosphorylated on Ser886 in a TOR-dependent manner (dotted arrow), and NOT4B was pulled down by LST8^36^. Chemical inhibitors of TOR and S6K1/2 used in this study are shown in blue. **b** Representative images of 12-day-old Col-0 and *not4* mutant seedlings grown on ½ MS medium for 6 days and then transferred to ½ MS supplemented with increasing concentrations of AZD8055. Scale bar = 1 cm. **c** Violin plot quantifying primary root growth following transfer to ½ MS+/- 5 μM AZD8055. Statistically significant differences were calculated by one-way ANOVA followed by Tukey’s test, presented in compact letter display (CLD) format. ns = not significant. **d** Violin plot quantifying primary root growth following transfer to ½ MS + /- 20 μM Torin. Statistically significant differences were calculated by one-way ANOVA followed by Tukey’s test, presented in compact letter display (CLD) format. ns = not significant. **e** Violin plot quantifying primary root growth of seedlings grown directly on ½ MS +/- 5 μM PF-4708671. Statistically significant differences were calculated by one-way ANOVA followed by Tukey’s test, presented in compact letter display (CLD) format. ns = not significant. **f** Steady state levels of total (RPS6) and phosphorylated (P-RPS6) RPS6A in 12-day-old Col-0 and *not4abc* seedlings treated for 0, 2 or 4 hours (**h**) with 1 μM AZD8055 (AZD). CBB = loading control. Approximate molecular weight in kDa is shown. **g** Average band intensity ratio of phosphorylated vs total RPS6 in Col-0 and *not4abc* seedlings based on three independent biological repeats. Inset graph shows the relative depletion of the P-RPS6/RPS6 ratio for Col-0 and *not4abc* compared to the untreated control (normalized to 100). Statistically significant differences were calculated by unpaired two-tailed Student’s t-test: *P < 0.05. All error bars in this figure represent ± SEM.

A recent large-scale phosphoproteomics study in Arabidopsis identified multiple novel downstream components of TORC1 signalling^37^. This included NOT4A, which was found to be directly phosphorylated on Ser886, and NOT4B, which was identified as a physical interactor of the broader TORC1 complex (for a simplified schematic of TOR signalling, including NOT4 proteins and drugs used in this study that target different nodes, see Fig. 4a). Treatment with the TOR inhibitors AZD8055 and rapamycin led to a  >4-fold decrease in NOT4A Ser886 phosphorylation, confirming its TOR-dependency^37^. A closer look at NOT4A-C using PTM Viewer 2.0^42^, which collates experimental evidence for post-translational protein modifications, revealed that all three NOT4 paralogs are in fact highly phosphorylated on multiple Ser residues (Supplementary Fig. 4). These confirmed phosphosites are primarily found downstream of the structured RING and RRM domains, in regions of the protein that are predicted by AlphaFold2^43^ to be disordered, further supported by independent bioinformatic analyses using IUPRED and PrDOS (Supplementary Fig. 4). Whilst NOT4A Ser886 is the only TOR-dependent phosphosite to be validated, the identification of NOT4B as a TORC1 interactor suggests that at least two, and probably all three, paralogs may be functionally associated with TORC1 signalling. In this context, each paralog may be phosphorylated at multiple sites, analogous to well-characterised TOR targets in animals such as S6K, 4E-BP1 and AKT1^29^.

Given this prior evidence linking NOT4 proteins to the TORC1 complex, and the requirement for co-translational QC to be coordinated with translational output, we explored the mechanistic connection between TOR and NOT4s. This was further motivated by the observation that loss of NOT4 function alters protein translation, including increased TOR activity (Fig. 2), and that sucrose supplementation (an established activator of TOR-dependent growth) restores wild-type root growth in *not4* mutants (Fig. 1h, i). We tested root growth inhibition of *not4* single, double, and triple mutant seedlings in response to increasing concentrations of the ATP-competitive TOR inhibitor AZD8055, which revealed that all lines except for *not4b* were hypersensitive relative to WT (Fig. 4b, c and Supplementary Fig. 5a). Treatment with the alternative inhibitor Torin confirmed that the enhanced sensitivity of both *not4a* and *not4c* is specific to direct inhibition of the TOR kinase (Fig. 4d and Supplementary Fig. 5b). In contrast, when we treated seedlings with PF-4708671, a specific inhibitor of the S6K branch of TORC1 signalling^44^, it was *not4b* that displayed the highest degree of sensitivity, indicating that isoform-specific subfunctions within the TOR signalling network may exist (Fig. 4e and Supplementary Fig. 5c). Furthermore, all mutant lines except the *not4b* single mutant exhibited elevated levels of phosphorylated RPS6 under control conditions (Supplementary Fig. 5d, e). Thus, despite elevated basal TOR activity, *not4* mutants were paradoxically hypersensitive to direct TOR inhibition, consistent with a partial uncoupling between TOR activation and the effective execution of TOR-dependent outputs.

Since exogenous sucrose was found to revert the growth phenotypes of *not4* mutants (Fig. 1h, i), we tested the effect of sucrose repletion on TOR activity. TOR was similarly induced in both *not4abc* and WT, increasing within 2 and 4 hours of 1% sucrose treatment and then decreasing again by 24 hours (Fig. Supplementary Fig. 5f), indicating that the capacity for TOR activation of RPS6 is not affected in the absence of NOT4s. Next, we tested the impact of AZD8055 on TOR activity. Despite hyperactive levels of TOR activity under control conditions (Fig. 2g, h), TOR was more sensitive to chemical shutdown in *not4abc* relative to WT, as evidenced by a faster relative depletion of the phosphorylated pool of RPS6 (Fig. 4f, g). This correlates with the heightened sensitivity of mutant seedlings to AZD8055 and Torin (Fig. 4b–e). Together, these findings indicate that the increased sensitivity of *not4* mutants to TOR inhibition does not arise from impaired TOR activation itself, but rather from defects in downstream TOR-dependent signalling outputs. This supports a model in which NOT4 function acts downstream of TOR to coordinate translational output with co-translational QC.

### The *not4abc* mutant transcriptome resembles TOR-inhibited wild-type

**Fig. 5:**
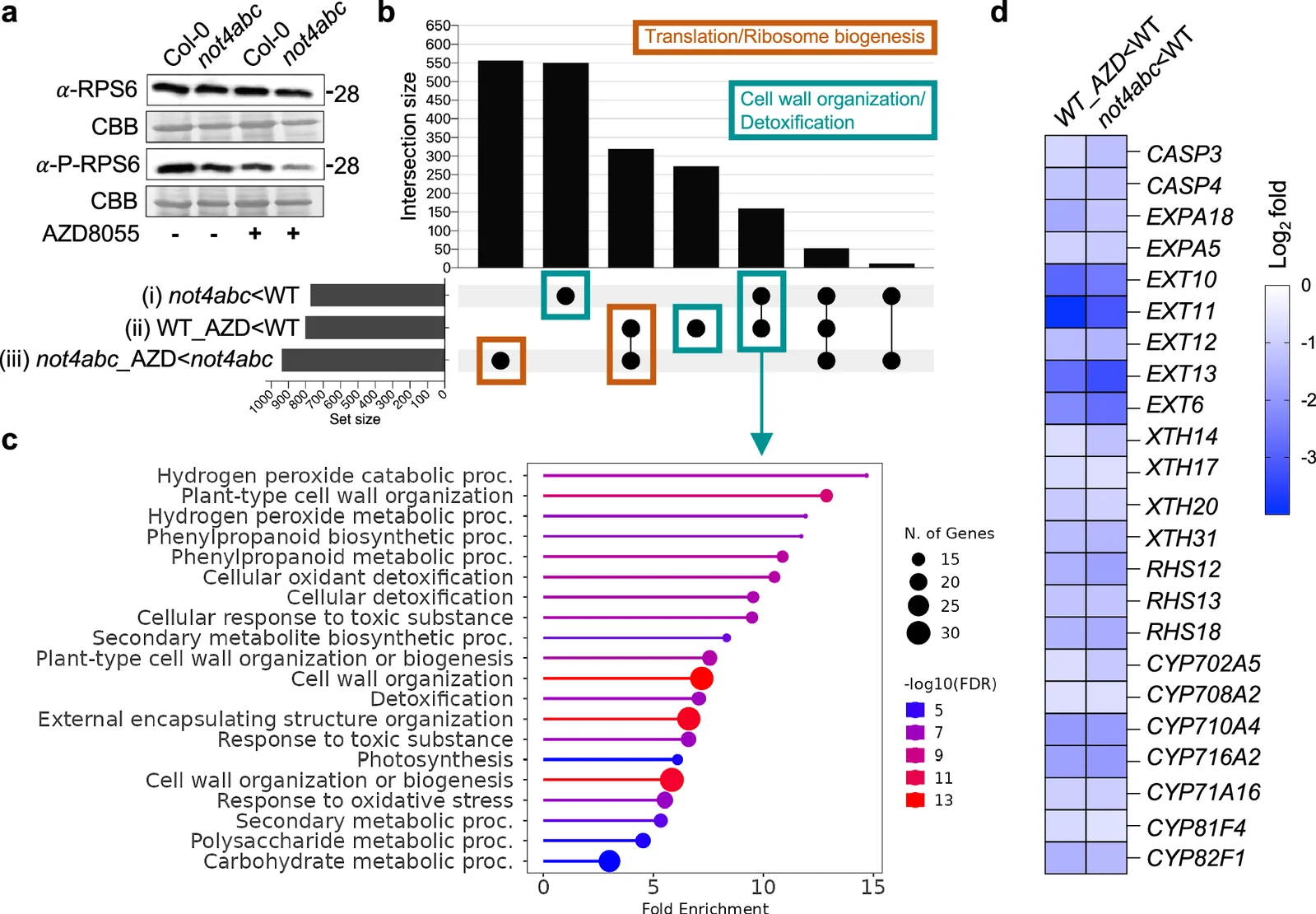
The *not4abc* transcriptome resembles that of TOR-inhibited wild-type. **a** Steady state levels of total (RPS6) and phosphorylated (P-RPS6) RPS6A in the 12-day-old Col-0 and *not4abc* seedlings treated for 0 or 2 hours (h) with 1 μM AZD8055 (AZD). A blot was conducted on total protein extracted from a subset of seedlings treated at the same time as those used for RNA-seq analysis and confirms the efficacy of the treatment for this specific experiment. CBB = loading control. Approximate molecular weight in kDa is shown. **b** UpSet plot showing set size and intersection size of significantly downregulated genes (1.5FC, padj <0.05) in (i) untreated *not4ab*c vs WT, (ii) AZD8055 treated vs untreated WT, (iii) AZD8055 treated vs untreated *not4abc*. Orange boxes denote intersecting gene lists that are enriched for GO terms linked to Translation/Ribosome biogenesis. Teal boxes denote intersecting gene lists that are enriched for GO terms linked to Cell wall organization/Detoxification etc. **c** Lollipop plot showing significant GO terms in the downregulated genes shared between untreated *not4ab*c vs WT and AZD8055-treated vs untreated WT. X-axis shows fold enrichment, circle size shows the number of genes, and colour denotes significance (-Log_10_ FDR). **d** Heat map showing Log_2_ changes of selected genes that are downregulated in both *not4ab*c vs WT and AZD8055-treated vs untreated WT.

The data presented in Fig. 4 reveal a functional interplay between NOT4 and TOR signalling. To further test whether loss of NOT4 function recapitulates TOR inhibition at the transcriptional level, we conducted an RNA-seq analysis of AZD8055-treated WT and *not4abc* seedlings, following a Western blot analysis to confirm efficacy of the AZD8055 treatment (Fig. 5a). Although several hundred genes were upregulated by AZD8055 in both lines, no significant GO terms were identified in individual or overlapping AZD8055-treated gene sets (Supplementary Fig. 6a and Supplementary Data 1). For the downregulated genes, a total of 805 and 941 genes were repressed by AZD8055 in WT and *not4abc*, respectively, with an overlap of 373 genes (Fig. 5b and Supplementary Fig. 6b). GO term analysis of this overlapping gene set revealed an enrichment of terms linked to translation and ribosome biogenesis, consistent with the expected effects of TOR inhibition on protein synthesis. The *not4abc*_AZD-unique gene set (568 genes) was also enriched for genes involved in translation. In contrast, the WT_AZD-unique gene set was largely made up of genes linked to cell wall structure/organisation and ROS catabolism and detoxification GO terms (Fig. 5c and Supplementary Fig. 6b). This was reminiscent of the downregulated genes in untreated *not4abc* vs WT (Fig. 2d). To directly assess this relationship, we compared the overlap in downregulated genes in *not4abc* vs WT and WT_AZD vs WT, which revealed 211 common genes (approximately 30% of the *not4abc* down genes). This shared gene set was enriched for similar GO terms linked to cell wall and detoxification (Fig. Supplementary Fig. 6c), including extensins, expansins, xyloglucans and diverse cytochrome p450 genes, as well as genes linked to Casparian strip formation (Fig. 5d). Together, these findings indicate that loss of NOT4 function partially phenocopies TOR inhibition at the transcriptional level, further supporting a requirement for NOT4s in the normal potentiation of TOR signalling outputs.

### Increased translation rates in *not4abc* provide resistance to *Pst* infection

**Fig. 6:**
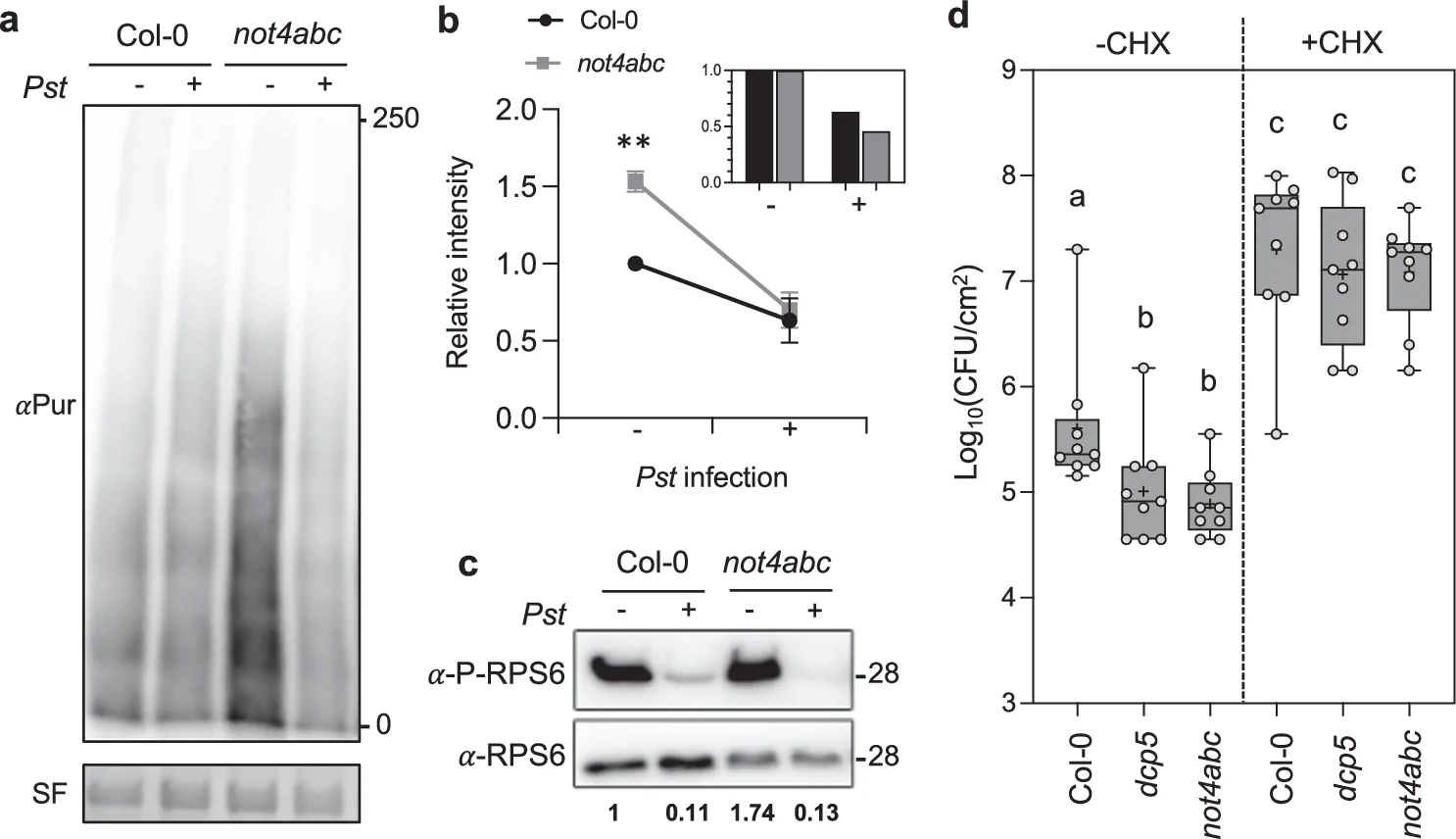
Increased resistance of *not4abc* to *Pseudomonas syringae pv. Tomato (Pst).* **a** anti-Puromycin Western blot of total protein extracted from 4-week-old *A. thaliana* leaves 24 hours after mesophyll-infiltration with either 10 mM MgCl_2_ mock (-) or 10^7^ CFU mL *Pst* (+). The large subunit of Rubisco visible through Stain-Free (SF) imaging serves as a loading control. **b** Quantification of puromycin incorporation in (**a**), based on three biological replicates (*n* = 3). Statistically significant differences were calculated by unpaired two-tailed Student’s *t*-test: **p < 0.01. **c** Steady state levels of total (RPS6) and phosphorylated (P-RPS6) Ribosomal Protein S6A (RPS6A) in 4-week-old *A. thaliana* leaves 24 hours after mesophyll-infiltration with either 10 mM MgCl_2_ mock (-) or 10^7^ CFU mL^-1^ *Pst* (+). **d** Bacterial population in 4-week-old *A. thaliana* leaves of wild type Col-0, *dcp5-1* and *not4abc* plants mesophyll-infiltrated with *Pst* solution at 10^4^ CFU mL^-1^+/− 10 μM CHX. Bacteria were extracted for counting three days post bacterial inoculation and CHX/DMSO treatment. Data are collated from two independent experiments. Different letters indicate statistical groups determined using unpaired two-tailed Student’s *t*-tests (*P* < 0.05). All error bars in this figure represent ± SEM.

To test the functional consequences of altered translational control under biologically relevant conditions, we analysed whether NOT4 function might be required for plant cell responses to non-pharmacological stresses that impact translation. A recent study showed that host protein synthesis is targeted by specific effectors secreted by *Pseudomonas syringae* pv*. tomato* (*Pst*), which dampen TOR activity and reduce translation rates to promote virulence^45^ This work showed that *dcp5* mutants, which lack DCP5, a component of the mRNA decapping complex that functions within processing bodies (P-bodies) and represses translation, are more resistant to *Pst*. We therefore tested how *not4abc* mutants, which have increased basal levels of TOR activity and translation rates reminiscent of those observed in *dcp5*^45^, might respond to *Pst* infection. We first examined how *Pst* affects translation rates (assessed by puromycin incorporation) and TOR activity (Fig. 6a–c). As reported earlier, *not4abc* seedlings exhibited higher basal translation rates and TOR activity; however, as observed following AZD8055 treatment (Fig. 4f, g), both parameters declined more rapidly in *not4abc* vs WT upon infection. Remarkably, *not4abc*, like *dcp5*, was significantly resistant to *Pst* infection, and this was linked to elevated translation in both mutants, as treatment with cycloheximide, a eukaryotic translational inhibitor, increased susceptibility to levels comparable with WT (Fig. 6d). Thus, elevated basal translational activity in the absence of NOT4 function may contribute to protection against a bacterial pathogen that suppresses host translation.

## Discussion

**Fig. 7:**
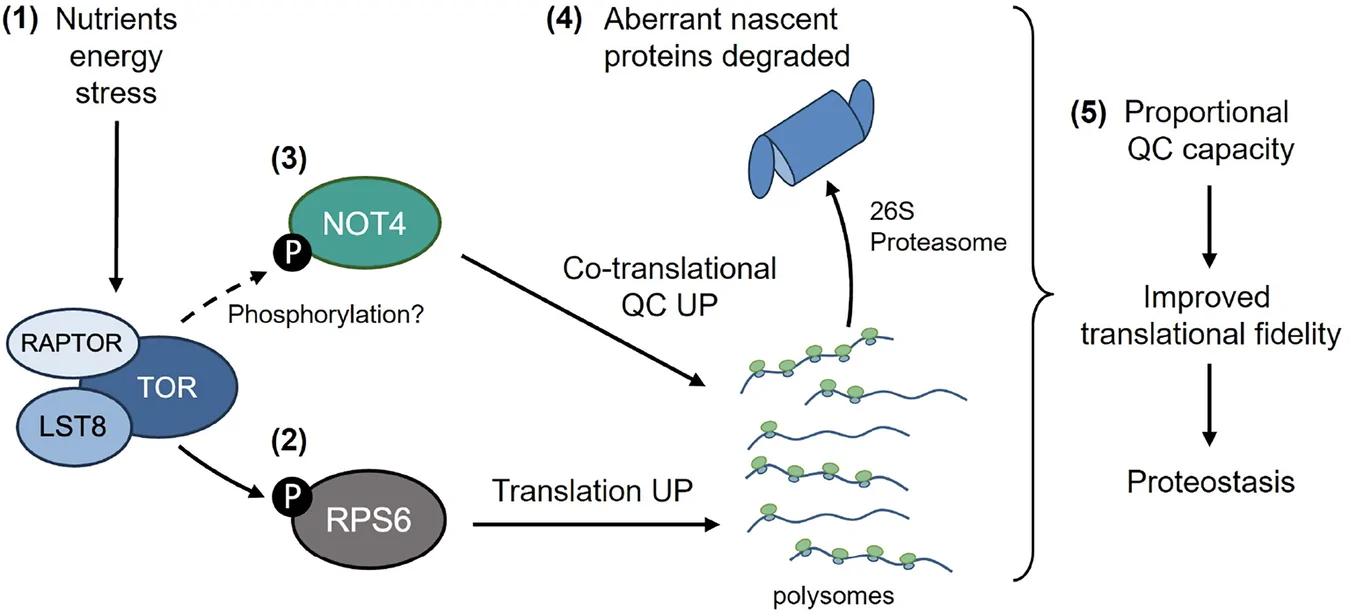
Proposed model for TOR–NOT4 coordination of translational output and co-translational quality control. (1) Environmental and physiological cues modulate TARGET OF RAPAMYCIN (TOR) signalling activity. (2) Active TOR promotes phosphorylation of translational regulators such as RPS6, thereby increasing global translational output. (3) TOR signalling functionally intersects with NOT4 proteins: NOT4s have been identified as TOR-regulated phosphotargets^37^, and the data presented here reveal a functional link between TOR activity and NOT4-dependent processes. (4) NOT4 E3 ubiquitin ligases regulate co-translational quality control by promoting the clearance of aberrant nascent polypeptides during translation. (5) Together, TOR-driven translation and TOR-NOT4 coupling may ensure that quality-control capacity is scaled with biosynthetic demand to maintain proteostasis across changing growth and stress conditions. Dashed arrows denote hypothesised regulatory connections inferred from prior work and from the integrated molecular, biochemical, and physiological evidence presented here.

Here we identify three NOT4-like E3 ubiquitin ligases in Arabidopsis and show they are important for regulating translation and mitigating proteotoxic stress. Loss of NOT4 function results in elevated TOR activity and translation rates (Fig. 2), increased accumulation of polyubiquitylated proteins (Fig. 3), and hypersensitivity to proteasome and TOR inhibition (Figs. 3, 4). Moreover, *not4* mutants exhibit growth phenotypes and a transcriptomic profile resembling that of TOR-inhibited wild-type plants (Figs. 1, 5), indicating that NOT4s are required for the normal manifestation of TOR-activated outputs. We propose that NOT4 function is coupled to TOR activity, enabling co-translational QC to be scaled in proportion to changes in translational output. In this model, NOT4-dependent QC pathways are increasingly engaged as TOR-driven translational output rises, thereby matching protein QC to biosynthetic demand (Fig. 7).

The identification of Arabidopsis NOT4s reveals evolutionary conservation of co-translational QC systems across eukaryotic kingdoms and expands the understanding of proteostasis regulation in plants^25^. In yeast and animals, RQC pathways rely on E3 ligases such as LTN1^4^ and HEL2^14^ to ubiquitylate stalled ribosomes and nascent peptides, promoting their clearance via the proteasome. In addition, the CCR4–NOT complex, with NOT4 as a key subunit, has emerged as a multifunctional node integrating mRNA surveillance, translational repression, and proteostasis^13,19–23^. Whether Arabidopsis NOT4s associate with the broader CCR4-NOT complex remains to be determined, though many of the core components of this holoenzyme are conserved^46–48^. The capacity for *At*NOT4s to partially compensate for loss of *Sc*NOT4 function in yeast (Fig. 1c) also supports this scenario, since yeast NOT4 is constitutively associated with the broader CCR4-NOT complex, whilst Drosophila and human NOT4 can also be catalytically active in isolation^49,50^. The inability of *At*NOT4C to reduce ScCOG1-GFP abundance relative to *At*NOT4A/B (Fig. 1c) may further reflect functional divergence linked to sequence variation outside the conserved RING and RRM domains. We also note that *At*NOT4s displayed differing electrophoretic mobilities relative to their predicted molecular weights, possibly due to post-translational modification and/or their extended intrinsically disordered regions^51^. Nonetheless, further observations support broader functional conservation between Arabidopsis NOT4 proteins and their counterparts in other organisms. Yeast *not4Δ* mutants accumulate nascent polyubiquitylated polypeptides and protein aggregates^7^, are hypersensitive to the protein misfolding elicitor AZC^21^, and exhibit increased translation rates due to de-repression of genes linked to the translational machinery^23^. Furthermore, *Sc*NOT4 has roles in promoting proteasome integrity via adaptor protein Ecm29, and *not4Δ* has weaker coupling of the 26S proteasome 19S Regulatory and 20S Core particles^22^. If this function is conserved in Arabidopsis, it could explain why *not4abc* has elevated caspase-like proteasome activity specifically (Fig. 3f), since weaker particle coupling could plausibly raise the apparent β1 caspase-like activity without affecting the β2 trypsin-like and β5 chymotrypsin-like proteasome activities. Future focus on identifying Arabidopsis NOT4 binding partners and ubiquitylation targets, particularly among factors linked to translational surveillance, proteostasis and proteasome function, will help to further delineate the extent to which plant NOT4 activity is conserved, and define if paralog-specific subfunctions exist, as hinted at in our work (e.g., Fig. 4c-e). Interestingly, although individual NOT4 paralogs displayed distinct sensitivities to TOR pathway inhibition, none of the corresponding single mutants exhibited enhanced sensitivity to proteasome inhibition (Supplementary Fig. 2e). This suggests that while individual NOT4 proteins contribute differentially to TOR signalling outputs, maintenance of proteostasis appears to be functionally buffered by redundancy between the NOT4 paralogs, with proteotoxic stress only emerging following combined loss of NOT4 function.

TOR is a master regulator of translation, ribosome biogenesis, and metabolism in response to nutrient and energy status^29,31^. Our work establishes a functional link between TOR activity and co-translational QC in plants, suggesting an evolutionary integration of growth-promoting pathways with QC mechanisms. The molecular and physiological data we present support a model in which TOR not only drives translational acceleration but simultaneously primes the proteostatic machinery to accommodate increased biosynthetic load and maintain proteome integrity. We propose this functional coupling could involve TOR-dependent phosphorylation of NOT4s, as has been previously documented for NOT4A^37^. Resolving whether TOR-dependent phosphorylation acts directly on NOT4s, indirectly via associated factors, or through multisite combinatorial modification will require targeted molecular dissection and represents an important direction for future investigation. Interestingly, NOT4 proteins in yeast and mammals are also known to be highly phosphorylated, particularly in the C-terminus^52–54^. Moreover, in yeast it was previously shown that certain mutants of the broader CCR4-NOT complex display hypersensitivity to TOR inhibition with rapamycin^55^. As such, it is plausible to speculate that this TOR-NOT4 signalling module may be conserved outside of the green lineage.

Disruption of specific NOT4 isoform combinations is associated with slow growth and reduced primary root length (Fig. 1), suggesting that the inability to clear aberrant protein products imposes a proteotoxic burden that suppresses downstream growth processes, and highlighting the importance of balancing translational throughput with proteome fidelity. These growth phenotypes are reminiscent of WT seedlings treated with TOR inhibitors (Fig. 4), and a previous study found that rapamycin-treated plants (sensitised to rapamycin through co-expression of a requisite yeast component) had altered cell wall structures, shorter roots, and reduced cell elongation^56^. Interestingly, we found that these growth defects are rescued through exogenous sucrose supplementation, but carbon starvation assays indicated that energy availability and mobilisation are not limited in *not4ab*c compared to WT. As such, these observations point to signalling rather than energy-deficit defects, consistent with perturbed TOR-dependent growth control. Notably, an analysis of TOR activity in *not4* mutants revealed paradoxical behaviours. Basal TOR activity was elevated relative to WT, but there was a faster depletion of the phosphorylated pool of RPS6 upon TOR inhibition with AZD8055. This indicates that NOT4s are dispensable for TOR activation and sugar sensing, and instead act predominantly downstream of TOR to enable the effective execution of TOR-dependent outputs. Consistent with this, our RNA-seq analysis revealed that the transcriptome of *not4abc* overlaps strongly with that of TOR-inhibited WT plants, including genes linked to nutrient transport and cell wall modification. Collectively, these data suggest that NOT4s integrate TOR-dependent signalling outputs with co-translational QC to ensure that increases in translational activity are matched by appropriate quality control, thereby maintaining translational homoeostasis, rather than acting as a core component of TOR activation itself. TOR-dependent signalling could provide one means of tuning this integration, for example by modulating protein-protein interactions that facilitate NOT4 engagement with ribosomes, protein complexes, or other QC factors proximal to TOR. Notably, it has been shown in yeast that NOT4 is required for the CCR4-NOT complex to support vacuolar V-ATPase stability and function, which is essential for nutrient-responsive TORC1 activation^55^.

These observations also raise the possibility that TOR signalling responds dynamically to compromised co-translational QC in the absence of NOT4 function. One interpretation consistent with our data is that loss of NOT4 function engages a compensatory feedback response onto TOR signalling. In this scenario, reduced co-translational QC in *not4* mutants is sensed by the cell, resulting in increased TOR activity (Fig. 2g, h) and transcriptional upregulation of *NOT4* genes (Supplementary Fig. 1d) as an attempt to restore protein QC. However, because functional NOT4 proteins are absent, elevated TOR activity cannot be effectively coupled to downstream QC or growth-related outputs. Importantly, this interpretation further supports a model in which NOT4s are not only regulated by TOR but are required for normal TOR-dependent outputs.

Finally, our data also suggest that elevated basal translation in *not4abc* may contribute to resistance during infection with *Pseudomonas syringae* pv. *tomato*, a pathogen known to suppress host translation to promote virulence. The resistance of *not4abc* plants, which maintain higher basal translation rates, mirrors that of *dcp5* mutants previously shown to counteract translation repression via impaired mRNA sequestration^45^. Notably, although *Pst* infection reduced translation rates in both Col-0 and *not4abc* at the sampled time point, altered basal translational homoeostasis prior to infection and during early infection stages may nevertheless contribute to the enhanced resistance phenotype observed in *not4abc*. Additional NOT4-dependent changes in proteostasis or stress signalling may also contribute. More broadly, TOR signalling has complex and context-dependent roles in plant immunity, with both positive and negative effects on disease resistance reported depending on the host-pathogen interaction and physiological context^57–60^. This suggests that maintaining active translation can be an important determinant of plant immune competence and implicates NOT4s indirectly in the dynamic interplay between translation control and pathogen defence. A robust assessment of *not4* mutant responses to diverse biotic and abiotic stresses that impact translation will help to further resolve the connection between translational flux and the capacity of co-translational QC systems to respond to it.

To conclude, our study identifies a functional connection between TOR signalling and NOT4-dependent co-translational QC that maintains signalling fidelity, coordinates co-translational QC with translational output to limit proteotoxicity, and modulates gene expression to control growth in Arabidopsis. As such, this work uncovers a new layer of regulation in plant TOR signalling that couples protein synthesis with co-translational quality surveillance to safeguard the proteome across varying translational demands, and which is potentially conserved across eukaryotes. Future studies will be essential to dissect the precise molecular events - such as substrate recognition and signalling dynamics - that underlie this coordination. Moreover, understanding how TOR-NOT4 signalling is tuned during development or in response to stress may open new avenues for manipulating proteostasis in crops, with important implications for growth, yield, and resilience in fluctuating environments.

## Methods

### Arabidopsis growth and transgenic lines

Seeds were sown either on a compost mixture (Levington F2:vermiculite: perlite in a 4:2:1 ratio) or on sterile half-strength Murashige and Skoog (MS) medium solidified with 0.8% agar, prepared with purified water and autoclaved for 15 min at 121 °C. Plants were cultivated in Weiss Technik Fitotron SGC 120 growth chambers under either long-day (16-h light/8-h dark, 22 °C) or short-day (12-h light/12-h dark, 22 °C) photoperiods.

The *prt6-1* and *dcp5* lines were described previously^61,62^. The *not4a* (GABI_134E03, GABI-KAT collection), *not4b* (SAIL_274_D03, SAIL collection) and *not4c* (SALK_093123, SALK collection) T-DNA insertion mutants were obtained from the European Arabidopsis Stock Centre (NASC). Homozygous insertions were identified by PCR using primers designed with T-DNA Express (primers listed in Supplementary Data 2), and loss of full-length transcript was confirmed by qPCR and RNA-seq (Fig. S1). Following the identification of an unrelated deletion affecting the *PGR3* locus in previously used material derived from the GABI_134E03 *not4a* line (see Acknowledgements), the *not4a* allele was re-isolated from the original NASC seed stock. Homozygous *not4a* plants were identified by PCR genotyping and subsequently backcrossed to the Col-0 wild-type background. Progeny were reselected to homozygosity for the *not4a* insertion, and the absence of additional deletions at the *PGR3* locus or neighbouring genomic regions was verified by PCR-based genotyping (primers listed in Supplementary Data 2) and RNA sequencing. All experiments reported here were performed using this independently re-isolated and validated *not4a* line. Double and triple *not4* mutant combinations were generated by genetic crossing.

To generate C-terminally GUS-tagged *NOT4A*, *NOT4B* and *NOT4C* driven by their native promoters, the full genomic DNA sequence (from approximately 2 kb upstream of the ATG to the stop codon) was amplified from seedling genomic DNA using attB-flanked primers (Supplementary Data 2) and recombined into pDONR201 using Gateway BP Clonase (Invitrogen; 11789020), prior to mobilisation into the destination binary vector pGWB533 using LR Clonase (Invitrogen; 11791100). For complementation of the *not4a* mutant, the *NOT4A* coding sequence lacking the stop codon was recombined into pDONR201 using Gateway BP Clonase II (Thermo Fisher Scientific) and subsequently transferred into the Gateway destination vector pEarleyGate103 using LR Clonase II to generate a C-terminal eGFP fusion driven by the CaMV 35S promoter (*35S::NOT4A-eGFP*). For all cloning primers, see Supplementary Data 2. Constructs were introduced into Agrobacterium tumefaciens strain GV3101 (pMP90) and transformed into Arabidopsis by the floral dip method. All lines were advanced to the T3 generation and homozygous lines were identified for phenotypic analysis.

### Phenotyping assays and seedling treatments

Flowering time was determined using 12–14 plants per genotype grown under both short- and long-day conditions. Plants were monitored daily, and once a bolt exceeded 1 cm, the number of rosette leaves and the corresponding day were recorded. Root growth assays were performed by incorporating chemicals directly into half-strength Murashige and Skoog (½ MS) medium (sucrose, PF-470867, tunicamycin) or by transient application in either solid or liquid media (AZD8055, TORIN1, puromycin). Sterilised seeds were plated and stratified for 48 hours, then grown for 12 days under long-day conditions (16-h light/8-h dark, 22 °C). For transient solid treatments, seedlings were first grown on ½ MS plates for 6 days and then aseptically transferred to fresh plates containing the indicated chemical concentrations: AZD8055 (1, 2, 5, 10 μM), TORIN1 (10, 15, 20 μM), PF-4708671 (1, 2, 5, 10, 15 μM), and tunicamycin (50, 75, 100 ng/ml). Root length was measured using ImageJ. For transient liquid treatments (AZD8055 activity assay, puromycin incorporation assay), seedlings were placed in wells containing liquid ½ MS medium supplemented with the appropriate chemical concentration. After the specified incubation period, seedlings were removed and processed for downstream analyses. For carbon starvation assays, seedlings were grown horizontally on ½ Murashige and Skoog (MS) medium for 10 days under long-day conditions. Plates were then transferred to complete darkness for the indicated time periods, after which seedlings were returned to standard light conditions for a 10-day recovery period prior to imaging and phenotypic assessment.

For Bortezomib phenotypic assays, seeds were sown on ½ MS plates (+/- 1% sucrose, containing DMSO, 3 µM BTZ or 4 µM BTZ. Seeds were plated and stratified for 48 hours, then grown for 12 days under long-day conditions (16-h light/8-h dark, 22 °C). Fresh weights were normalized to those seedlings grown on control DMSO plates.

### Pst infection assays

*Pseudomonas syringae* pathovar *tomato* (*Pst*) wild-type strain DC3000 was grown in King’s B medium supplemented with 100 mg/L rifampicin at 28 °C. Overnight *Pst* cultures were centrifuged for 7 minutes at 1500 × *g*. Pellets were washed twice with 10 mM MgCl_2_ prior to measuring the optical density at 600 nm (OD_600_). Bacterial inocula were infiltrated with a needleless syringe into the abaxial side of fully developed leaves from 4-week-old *A. thaliana* plants. For puromycin incorporation and TOR activity assays, *Pst* inocula were prepared at a final OD_600_ of 0.1 in 10 mM MgCl_2_. Two leaf discs (6 mm diameter) per sample were harvested 24 hours post-inoculation and, for puromycin incorporation assays, additionally treated with 50 µM puromycin in liquid ½ MS medium for 1 hour prior to protein extraction and immunoblotting. For bacterial growth assays, *Pst* inocula were prepared at a final OD_600_ of 0.0001 in 10 mM MgCl_2_ supplemented with 10 μM CHX or an equivalent volume of DMSO. Bacterial populations were estimated three days after inoculation by plating serial dilutions of bacteria extracted from two 6-mm-diameter leaf discs in 200 µL 10 mM MgCl_2_, homogenized with a tissue lyser (Retsch). Colony-forming units were manually counted 48 hours after plating.

### RNA-sequencing

RNA was isolated from 12-day-old seedlings for all sequencing experiments. Library construction, quality control, and sequencing were performed by Novogene UK Ltd. RNA integrity was confirmed by agarose gel electrophoresis (28S and 18S rRNA bands), NanoDrop quantification, and Agilent 2100 Bioanalyzer analysis. mRNA was purified using poly-T oligo-attached magnetic beads, fragmented, and reverse transcribed into first-strand cDNA with random hexamer primers, followed by second-strand cDNA synthesis using either dUTP (directional library) or dTTP (non-directional library)^63^. Libraries were quantified with a Qubit Fluorometer (Invitrogen), validated by qPCR and Bioanalyzer, pooled, and sequenced on the Illumina NovaSeq 6000 platform. Raw paired-end reads were processed with in-house Perl scripts to remove low-quality reads, reads containing poly-N, and adapter contamination. Clean reads were aligned to the *Arabidopsis thaliana* reference genome (TAIR10) using HISAT2 v2.0.5^64^. Transcript assembly was performed with StringTie v1.3.3b^65^, and read counts were generated with FeatureCounts v1.5.0-p3^66^. Gene expression levels were calculated as FPKM (Fragments Per Kilobase of transcript per Million mapped reads). Differential expression analysis between two conditions (three biological replicates each) was conducted using DESeq2 v1.20.0^67^. *P*-values were adjusted for multiple testing with the Benjamini–Hochberg method^68^. Genes with adjusted *P*-values ≤ 0.05 and an absolute fold change ≥ 2 were defined as differentially expressed, except for the *not4abc* versus Col-0 comparison, for which an absolute fold-change threshold of ≥ 1.5 was applied. Gene Ontology (GO) enrichment of differentially expressed genes was performed using the topGO R package^69^, with significance defined as *p*  <  0.05^70^. KEGG pathway enrichment was assessed with the clusterProfiler R package^71^. KEGG provides a curated resource for mapping high-level biological functions from genome-wide datasets (http://www.genome.jp/kegg/).

### Functional enrichment and data visualisation

Differentially expressed genes (DEGs) identified from RNA-seq analysis were subjected to Gene Ontology (GO) enrichment analysis using ShinyGO v0.85.1 (https://bioinformatics.sdstate.edu/go/). Enrichment analysis was performed using default parameters, and significantly enriched GO terms were identified based on a false discovery rate (FDR)-corrected *P*-value cutoff of 0.05. Lollipop plots were generated directly within ShinyGO to visualise enriched GO categories. Venn diagrams were generated using Venny 2.0 (https://bioinfogp.cnb.csic.es/tools/venny/index2.0.2.html) to illustrate overlaps among DEG sets from different experimental comparisons. Volcano plots were generated using VolcaNoseR (https://huygens.science.uva.nl/VolcaNoseR/), enabling interactive visualisation of gene expression changes based on log₂ fold change and statistical significance. UpSet plots were generated using Chiplot (https://www.chiplot.online/upset_plot.html) to visualise intersections among multiple DEG sets. Default parameters were used for all online tools unless otherwise specified.

### qRT-PCR

Arabidopsis seedlings were flash-frozen in liquid nitrogen and ground to a fine powder. Total RNA was extracted using the RNeasy Plant Mini Kit (Qiagen) following the manufacturer’s instructions and quantified with a NanoDrop 1000 spectrophotometer (Thermo Scientific). First-strand cDNA was synthesized using SuperScript II Reverse Transcriptase (Thermo Fisher Scientific). Quantitative PCR (qPCR) primers were designed using NCBI Primer-BLAST^72^ and verified to span exon–exon junctions (primers listed in Supplementary Data 2**)**. Primer specificity was confirmed through melt-curve analysis. Relative transcript abundance was determined by the ΔCt method^73^, using ACTIN7 and/or PP2A as internal reference genes. qPCR reactions were prepared with Brilliant III Ultra-Fast SYBR Green QPCR Master Mix with Low ROX (Agilent) using 10 ng cDNA per reaction. Amplifications were carried out on either an AriaMx Real-Time PCR System (Agilent) for 96-well assays or a QuantStudio 5 system (Thermo Fisher Scientific) for 384-well assays. Negative controls without template were included in all experiments to confirm absence of genomic DNA contamination.

### Protein extractions and Western Blotting

Total protein was extracted from 12-day-old *Arabidopsis thaliana* seedlings. Tissue was homogenized to a fine powder in liquid nitrogen using a TissueLyser II (QIAGEN) and resuspended in extraction buffer (150 mM Tris-HCl pH 7.0, 150 mM NaCl, 5 mM EDTA, 2 mM EGTA, 5% (v/v)glycerol, 1% (v/v) Triton X-100, 0.5% (v/v) SDS, 10 mM DTT, and cOmplete™ Mini EDTA-free Protease Inhibitor Cocktail (Roche; 1 tablet per 10 ml)) at a ratio of 1:4 (w/v). Lysates were clarified by centrifugation at 21,130 × g for 30 min at 4 °C, and supernatants were collected as crude protein extracts. For SDS–PAGE, samples were prepared in 4× loading buffer (120 mM Tris-HCl pH 6.8, 20% glycerol, 10% β-mercaptoethanol, 4% SDS, 0.02% bromophenol blue) and separated on 10% polyacrylamide gels (resolving gel: 0.38 M Tris-HCl pH 8.8, 10% (w/v) acrylamide, 0.1% (w/v) SDS, 0.05% (w/v) APS, 0.07% TEMED; stacking gel: 132 mM Tris-HCl pH 6.8, 4% (w/v) acrylamide, 0.1% (w/v) SDS, 0.05% (w/v) APS, 0.15% (v/v) TEMED) using the BIORAD Mini-PROTEAN system. Proteins were transferred to PVDF membranes overnight at 4 °C. Membranes were blocked with 5% semi-skimmed milk in TBST (20 mM Tris-HCl pH 7.5, 150 mM NaCl, 0.1% Tween-20) for 1 h and incubated with primary antibodies diluted in 5% milk-TBST: anti-RPS6 (1:2000; Agrisera, AS19 4292), anti-RPS6-P (1:2000; Agrisera, AS19 4291), anti-UBCJ2 (1:5000; Enzo Life Sciences, BML-PW8810-0500), anti-GFP/YFP (1: 1000; Roche 11814460001), 1:4000 anti-HA (1:4000; Sigma-Aldrich H3663), anti-ubiquitin (1:1000, Agrisera AS08307A), anti-RPN10 (1:1000, Agrisera, AS19 4266), anti-RPT4a (1:3000, Agrisera, AS19 4263), and anti-Puromycin (1:10,000; Merck, MABE343). Incubation was performed for ≥1 h (up to overnight) at 4 °C. Membranes were washed three times (5 min each) in TBST and probed with HRP-conjugated secondary antibodies, anti-rabbit (1:10,000; Cell Signaling Technology, 7074) or anti-mouse (1:10,000; Sigma, A5278), for 1 h. Following three additional washes in 1x TBST, membranes were developed using Pierce™ ECL Western Blotting Substrate (Thermo Scientific, 32106) for 1 min and exposed to X-ray film (FUJIFILM SUPER RX) using a HI-SPEED-X intensifying screen. Films were developed with an Xograph Compact X4 Automated Processor and imaged on a light box with a Nikon D40 SLR camera. Unless otherwise stated, immunoblotting experiments were repeated independently three or more times with similar results.

### TOR activity assay

Twelve-day-old seedlings were incubated in liquid ½ MS medium containing 1 µM AZD8055 for 0, 2, or 4 hours. Total protein was extracted as described above, and TOR activity was assessed by immunoblotting using anti-RPS6 and anti-RPS6-P antibodies. TOR activity was quantified as the ratio of phosphorylated RPS6 (RPS6-P) to total RPS6.

### Puromycin incorporation assay

Twelve-day-old seedlings were treated with 50 µM puromycin in liquid ½ MS medium for 0, 20, or 60 min. Following the 60-min incubation, seedlings were transferred to fresh medium containing either 50 µM cycloheximide (CHX) or an equivalent volume of DMSO and incubated for a further 60 min. Total protein was extracted, and puromycin incorporation was assessed by immunoblotting with anti-puromycin antibody.

### Proteasome activity assays

Proteasome activity measurements in Arabidopsis were performed using the Proteasome-Glo™ Cell-Based Assay from Promega, Walldorf (cat. no. G1180)^74^. Briefly, seedlings of the indicated genotypes were homogenized in 600 μl ice cold proteasome extraction buffer (50 mM HEPES-KOH pH 7.2, 2 mM DTT, 2 mM ATP, 0.25 M Sucrose) and incubated on ice for 10 minutes. Subsequently, samples were centrifuged at 4 °C and 17,000 x g for 10 minutes. During centrifugation, proteasome assay reagents were prepared using Suc-LLVY-Glo™, Z-LRR-Glo™ and Z-nLPnLD-Glo™ substrates to measure chymotrypsin-like, trypsin-like and caspase-like proteasome activities, respectively, according to the manufacturer’s instructions. After centrifugation, 150 μl of proteasome extract supernatants were transferred to a new microcentrifuge tube and diluted 1:1 with ice-cold proteasome extraction buffer. 50 μl of each diluted extract was pipetted into three different wells of a white 96-well plate to be able to measure the three different protease activities (chymotrypsin-like, trypsin-like and caspase-like) individually. After incubating the plate for 15 minutes at room temperature in darkness, 50 μl of the respective substrate solutions were added to the proteasome extracts using a multichannel pipette to start the reaction. Luminescence was measured in a microplate reader (Tecan Infinite 200 PRO) over a time course of 30 minutes with data points taken every minute. Proteasome activities were determined from the rate of change in luminescence over time and are presented as relative luminescence units per minute (RLU min-1).

### Quantification of immunoblots using ImageJ

Relative protein abundance was quantified using ImageJ (NIH, USA; https://imagej.nih.gov/ij/). Western blot films were scanned at 600 dpi in TIFF format, and raw image files were preserved. Duplicate JPEG copies were converted to grayscale for analysis. Rectangular regions of interest (ROIs) were drawn around each protein band, with identical ROI sizes applied across all lanes for a given blot. Background ROIs were defined in adjacent unstained regions. Mean grey values were measured using the “Measure” function, and pixel intensities were inverted (255 – X). Net band intensity was calculated as signal minus background. Protein abundance was normalized to the corresponding loading control in each lane by calculating the ratio of net band intensity to net loading control intensity.

### Histochemical GUS staining

7-day-old seedlings were incubated in GUS staining solution consisting of 0.1 M PBS (pH 7.0), 2 mM X-Gluc, 0.1% (v/v) Triton X-100, supplemented with 1% (w/v) potassium ferricyanide and 1% (w/v) potassium ferrocyanide. Approximately 1 ml of staining solution was added per sample, and seedlings were kept in darkness at 37 °C for 24 hours. Following incubation, the staining solution was replaced with 1 ml of fixative (ethanol:acetic acid, 3:1, with 1% (v/v) Tween-20). Samples were gently agitated at room temperature, with repeated replacement of fixative until tissues were transparent. Cleared samples were rinsed briefly in sterile distilled water and mounted on microscope slides in 50% (v/v) glycerol. Images were captured using a bifocal light microscope with Touplite software. Unless otherwise stated, GUS staining was repeated independently three times with similar results.

### Protein structure prediction

Predicted protein structures for NOT4A, NOT4B, and NOT4C were generated using AlphaFold2^43^ with default parameters via the AlphaFold Protein Structure Database^75^, and visualised using PyMOL. Regions of intrinsic disorder were inferred from low pLDDT confidence scores and independently assessed using the disorder prediction algorithms IUPred2A^76^ and PrDOS^77^, which yielded highly consistent predictions. These analyses were used for qualitative interpretation only.

### Yeast assays

To generate C-terminally HA-tagged constructs, the coding sequences of *Saccharomyces cerevisiae NOT4* (*ScNOT4*) and *Arabidopsis thaliana NOT4A*, *NOT4B*, and *NOT4C* were amplified by PCR from yeast or seedling cDNA (primers listed in Supplementary Data 2), respectively. cDNA was synthesised from total RNA using SuperScript II reverse transcriptase (Thermo Fisher Scientific, Invitrogen; Cat. No. 18064-014). Following ligation into either pD-TOPO (NOT4A and NOT4B) or pDONR221 (NOT4C), PCR products were mobilised into the Gateway-compatible yeast expression vector pAG413 GPD-CT-HA using LR Clonase (Invitrogen; 11791100). The *ScNOT4* L35A RING mutation was generated by site-directed mutagenesis. For C-terminally GFP-tagged COG1, the coding sequence was amplified, ligated into pD-TOPO and mobilised into pAG416 GPD-CT-GFP using LR Clonase (Invitrogen; 11791100). The *not4Δ* (*yer068wΔ::kanMX*) strain was maintained on medium supplemented with G418 (200 μg mL⁻¹). Transformation into *not4Δ* was performed using the lithium acetate method. Briefly, a loop of cells was mixed with 2 μg of plasmid DNA and transformation buffer (33% (v/v) polyethylene glycol 3350, 0.33 M lithium acetate, 0.66% (v/v) β-mercaptoethanol), incubated at 37 °C for 45 min with shaking (200 rpm), and plated on synthetic drop-out medium (Formedium). Plates were incubated at 30 °C, with G418 included where required to maintain selection for the *not4Δ* background. Total protein was extracted by alkaline lysis. Cell pellets were resuspended in extraction buffer (0.1 M NaOH, 50 mM EDTA, 2% (v/v) β-mercaptoethanol, 2% (v/v) SDS) and heated at 90 °C for 20 min, with 3 M acetic acid added halfway through incubation. Sample buffer (250 mM Tris-HCl (pH 6.8), 50% (v/v) glycerol, 0.05% bromophenol blue) was added, samples were clarified by centrifugation, and supernatants were used in downstream Western blotting.

### Reporting summary

Further information on research design is available in the Nature Portfolio Reporting Summary linked to this article.

## Supplementary information

Supplementary Information

Description of Additional Supplementary Files

Supplementary Data 1

Supplementary Data 2

Reporting Summary

Transparent Peer Review file

## Source data

Source Data

## Data Availability

The raw RNA-seq data generated in this study have been deposited in the Gene Expression Omnibus (GEO) under accession code GSE319908. Complete differential expression tables for all RNA-seq comparisons are provided in the Supplementary Information. Source data are provided with this paper.
