# Supplementary material for: TOR–NOT4 signalling couples translation to co-translational quality control in plants
Source: Nat Commun. 2026 Aug 24;17:10101. doi: 10.1038/s41467-026-77200-0 (PMC13598129; doi:10.1038/s41467-026-77200-0)
Supplement: Supplementary file 1 [file 41467_2026_77200_MOESM1_ESM.pdf]

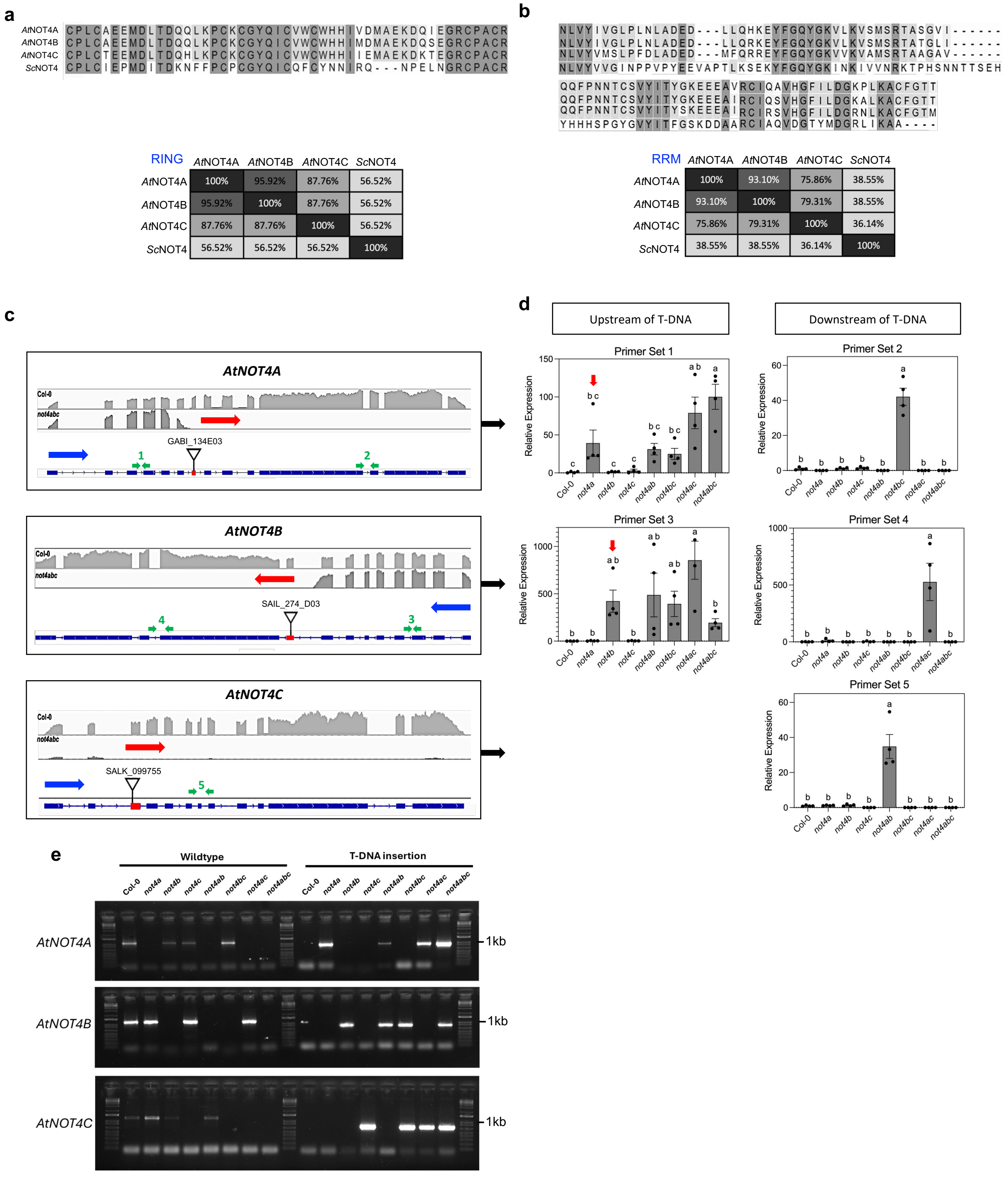

Supplementary Figure 1. Identification and phenotypic characterization of *Arabidopsis* NOT4-like proteins, related to main Figure 1. (**a, b**) Amino acid residue alignment and sequence identity matrix for the RING domain (a) and RRM domain (b) of Arabidopsis (*At*) and yeast (*Sc*) NOT4 orthologs. (**c**) Gene schematics showing introns/exons, T-DNA insertion sites (red boxes), primer positions (green arrows) and direction of transcription (blue arrows) for *AtNOT4A*, *AtNOT4B*, and *AtNOT4C*. The mapped reads from the Col-0 and *not4abc* RNA-seq data (main Fig. 2) were visualised in IGV and are shown in grey, illustrating the lack of transcript reads downstream of each respective T-DNA insertion, thus confirming gene knock out. (**d**) RT-qPCR of *NOT4A-C* in different *not4* single and combination mutants, using primers upstream and downstream of the T-DNA insertion (primers denoted in c). Individual data points represent n = 4 biological replicates. Error bars represent ± SEM**.** Red arrows show enhanced expression of the 5' region of each *NOT4* gene upstream of the T-DNA insert in that same gene. (**e**) Genotyping PCR confirming homozygous T-DNA insertions in *not4* single, double and triple mutant.

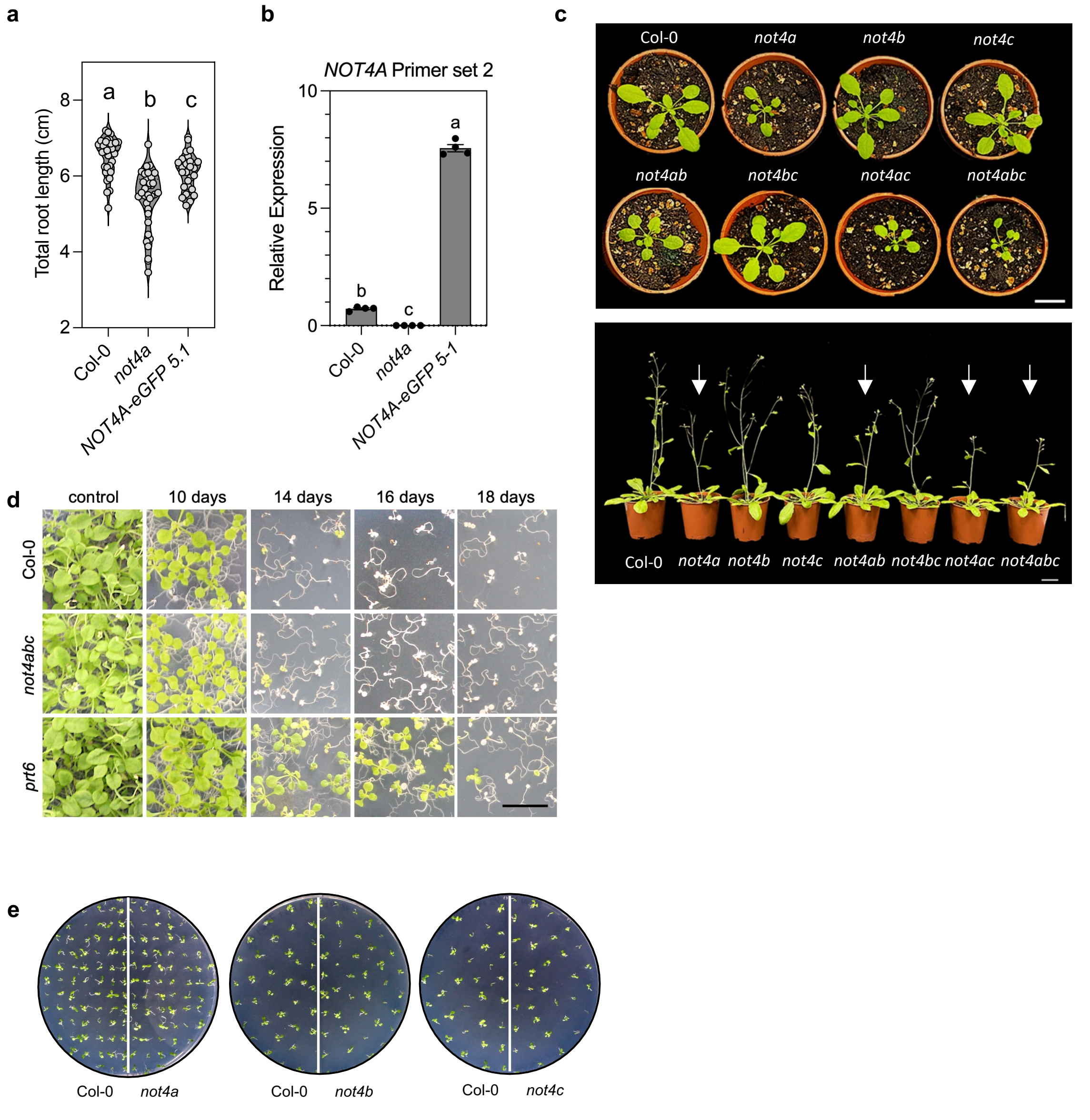

Supplementary Figure 2, Phenotypic assessments, related to main Figures 1 and 3. (**a**) Primary root length of 7-day-old Col-0, *not4a*, and *35S::NOT4A-eGFP/not4a* seedlings grown on ½ MS medium. Different letters indicate statistically significant differences determined by one-way ANOVA followed by Tukey's multiple comparison test. (**b**) RT-qPCR analysis confirming expression of *NOT4A* in the *35S::NOT4A-YFP/not4a* complementation line. Expression was normalised to *ACTIN2* and is shown relative to Col-0. Data represent mean ± SEM from n = 4 biological replicates. **(c)** Representative rosette and bolting phenotypes for *not4* single and combination mutants. White arrows highlight genotypes with temporally delayed flowering (quantified in main figure 1j). Scale bar = 2 cm(**d**) Representative images of seedlings grown for 5 days then transferred to the dark for 10-18 days and then transferred back into the light for 7 days. *prt6* is resistant to extended darkness, as denoted by its capacity for greening after dark. Col-0 and *not4abc* appear equally sensitive. Scale bar = 1 cm. (**e**) Representative images of 14-day-old seedlings treated with 4 μM Bortezomib. Assays in c, d and e are representative of experiments conducted three times with similar results.

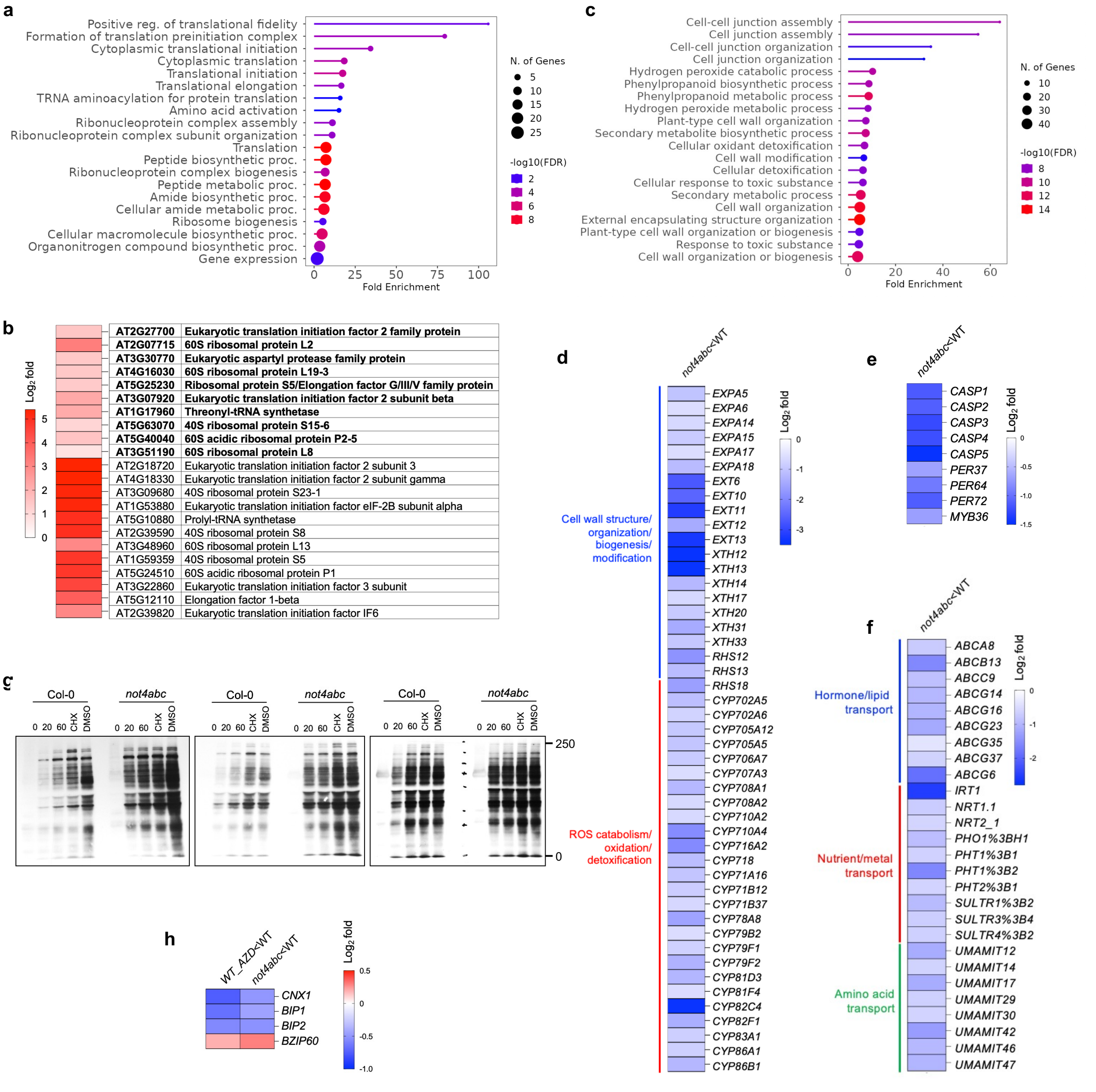

Supplementary Figure 3. RNA-seq analysis reveals elevated translation rates in *not4abc*, related to main Figures 2 and 3. (**a**) Lollipop plot showing significant GO terms in the *not4abc* upregulated DEGs (>2 FC, P < 0.05). X-axis shows fold enrichment, circle size shows number of genes, and colour denotes significance (-$\log_{10}$ FDR). **(b)** Heat map showing $\log_2$ fold change of *not4abc* upregulated genes linked to protein translation GO terms in (a). Genes in bold are those that have padj < 0.05 (see main Figure 2d), and non-bold genes are those that have P < 0.05. (**c**) Lollipop plot showing significant GO terms in the *not4abc* downregulated DEGs (>2 FC, P < 0.05). X-axis shows fold enrichment, circle size shows number of genes, and colour denotes significance (-$\log_{10}$ FDR). (**d-f**) Heat maps showing log2 fold change of select *not4abc* downregulated genes linked to cell wall structure/modification and ROS catabolism (d), Casparian strip formation (e) and hormone, nutrient, metal and amino acid transport (f). (**g**) Three independent biological repeats of the puromycin incorporation assay in main figure 2e used for quantification in main figure 2f. The first blot shown here is the same as in figure 2e. (**h**) Heat map showing $\log_2$ fold change of UPR genes in the denoted RNA-seq comparisons.

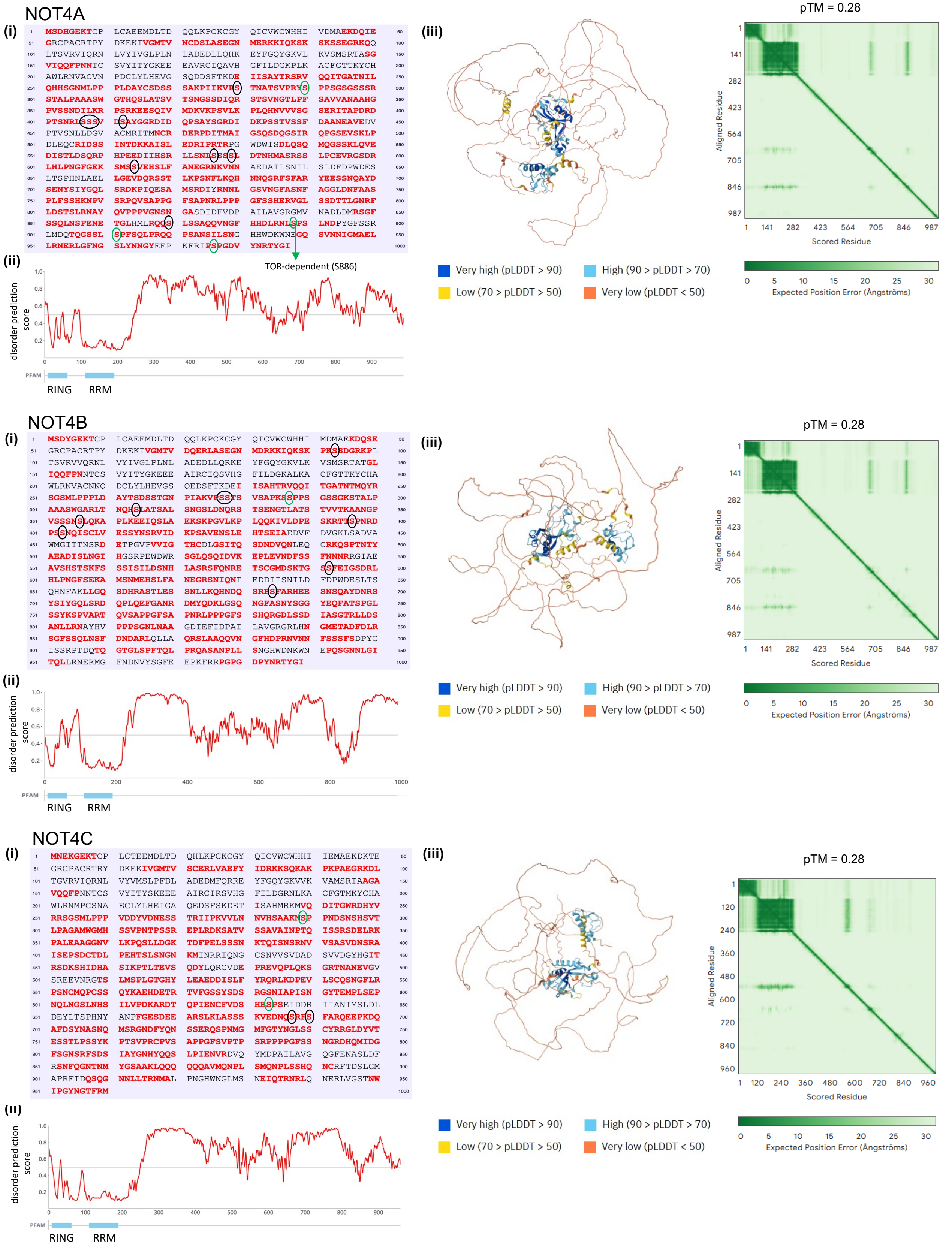

Supplementary Figure 4. Structural predictions and experimentally validated phosphorylation sites in Arabidopsis NOT4 proteins. For each NOT4 paralog: (**i**) amino acid sequence of full-length protein. Black and red amino acid letters denote ordered vs disordered amino acid residues, respectively (as predicted by PrDOS). Black circles denote S/T residues that have been identified as phosphorylated (based on PTM viewer 2.0). Green circles denote validated phosphorylated serines that immediately precede a proline residue (a preferred context for TOR phosphorylation). The TOR-dependent phosphosite (van Leene *et al.* 2019) in NOT4A is shown. (**ii**) IUPred2A output, which predicts disorder based on energy estimation, identifying regions likely to be unstructured. The red line is the disorder prediction score, and the horizontal line represents the confidence threshold, with values above the line indicating predicted disorder. RING and RRM domains predicted by PFAM are shown to be ordered. (**iii**) Full-length AlphaFold2 predictions show high per-residue confidence (pLDDT) within the N-terminal RING and RRM domains, followed by a largely disordered C-terminal region, leading to a low overall predicted template modelling (pTM) score of 0.28 for each isoform.

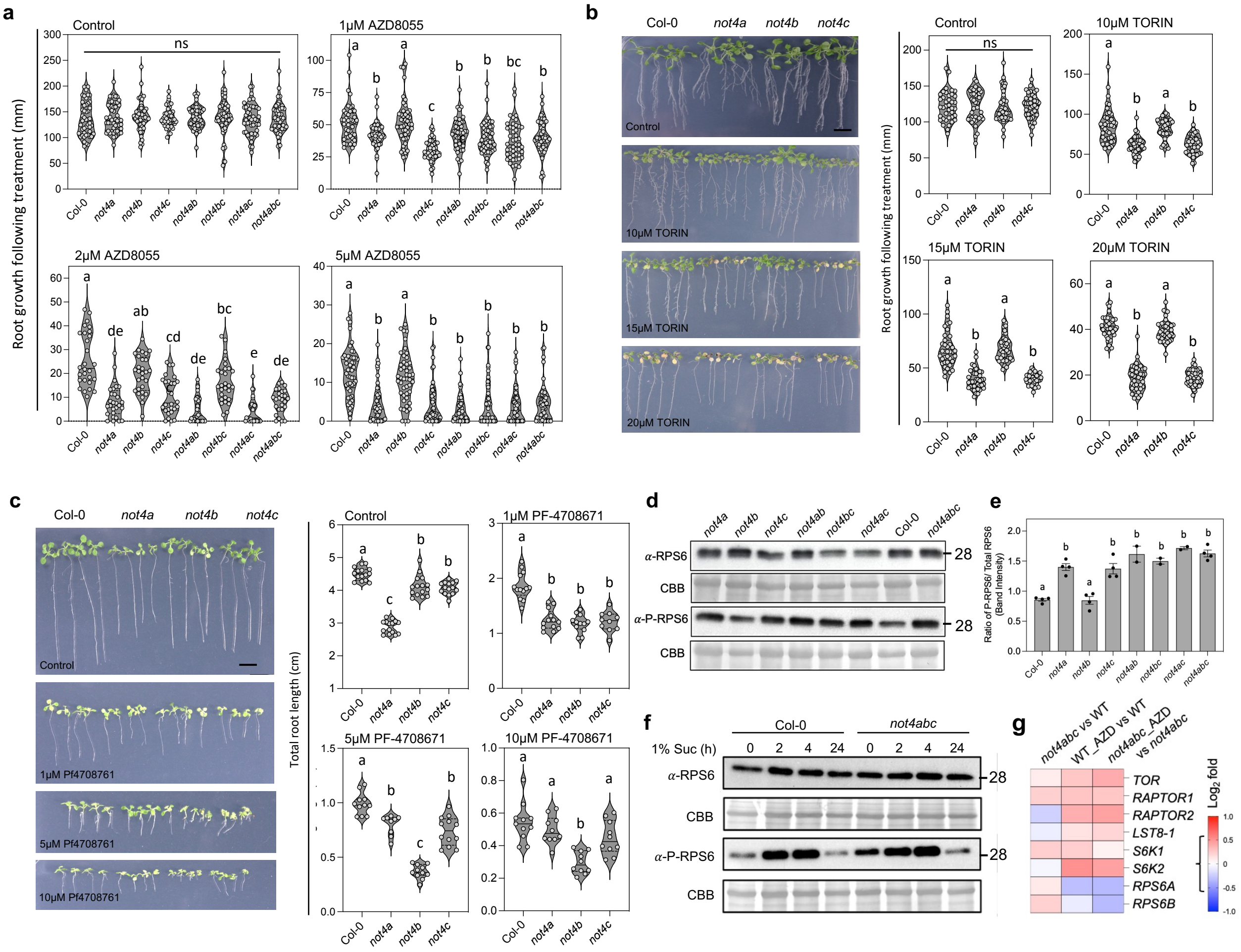

Supplementary Figure 5. Loss of NOT4 activity leads to TOR signalling defects, related to main Figure 4. (**a**) Violin plots quantifying primary root growth following transfer to ½ MS ± various concentrations of AZD8055. Statistically significant differences were calculated by one-way ANOVA followed by Tukey's test, presented in compact letter display (CLD) format. ns = not significant. (**b**) Representative images and corresponding violin plots quantifying primary root length of 12-day-old Col-0 and *not4* mutant seedlings grown on ½ MS medium for 6 days and then transferred to ½ MS supplemented with increasing concentrations of Torin. Scale bar = 1 cm. Statistically significant differences were calculated by one-way ANOVA followed by Tukey's test, presented in compact letter display (CLD) format. ns = not significant. (**c**) Representative images and corresponding violin plots quantifying primary root length of 12-day-old Col-0 and *not4* mutant seedlings grown directly on ½ MS medium ± Pf4708761. Scale bar = 1 cm. Statistically significant differences were calculated by one-way ANOVA followed by Tukey's test, presented in compact letter display (CLD) format. ns = not significant. (**d**) Steady-state levels of total (RPS6) and phosphorylated (P-RPS6) Ribosomal Protein S6A (RPS6A) in 12-day-old Col-0 and *not4* seedlings. CBB loading control. Approximate molecular weight in kDa is shown. The Col-0 and *not4bc* portion of the blot is repeated in main figure 2g. (**e**) Average quantified ratio of P-RPS6 to RPS6 calculated from 2-4 independent biological replicates (n = 2-4). Value is shown within each bar. Statistically significant differences were calculated by one-way ANOVA followed by Tukey's test, presented in compact letter display (CLD) format. (**f**) Steady-state levels of total (RPS6) and phosphorylated (P-RPS6) Ribosomal Protein S6A (RPS6A) in 12-day-old Col-0 and *not4abc* seedlings treated with 1% sucrose for denoted times in hours (h). CBB loading control. Approximate molecular weight in kDa is shown. (**g**) Heat map showing log2 fold change of TOR-signalling-associated genes in the denoted RNA-seq comparisons. Note that in *not4abc* vs WT, there is no significant up- or downregulation.

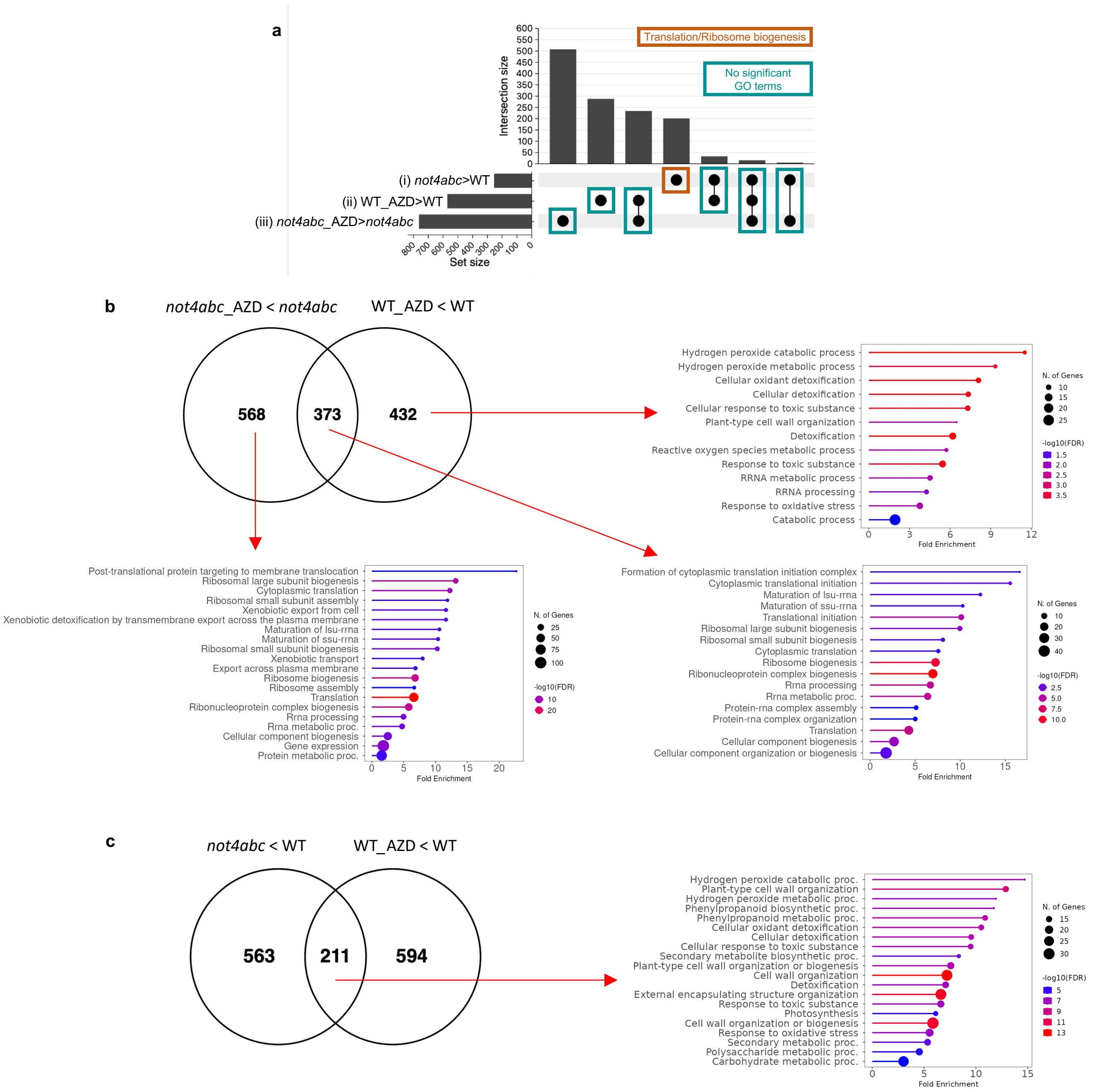

Supplementary Figure 6. RNA-seq and GO term analyses, related to main Figure 5. (a) UpSet plot showing set size and intersection size of significantly upregulated genes (>1.5 FC, padj < 0.05) in (i) untreated *not4abc* vs WT, (ii) AZD8055-treated vs untreated WT, and (iii) AZD8055-treated vs untreated *not4abc*. No significant GO terms are associated with the intersect gene lists. (**b, c**) Venn diagram showing downregulated gene set overlaps in the designated RNA-seq comparisons. Arrows call out GO terms linked to the intersect gene lists.
