# Supplementary material for: TOR–NOT4 signalling couples translation to co-translational quality control in plants
Source: Nat Commun. 2026 Aug 24;17:10101. doi: 10.1038/s41467-026-77200-0 (PMC13598129; doi:10.1038/s41467-026-77200-0)
Supplement: Supplementary file 2 [file 41467_2026_77200_MOESM2_ESM.pdf]

Description of Additional Supplementary Files

Supplementary Data 1: Differentially expressed genes, GO term enrichment and associated volcano and UpSet plot analyses.

Supplementary Data 2: List of primers used in this study
