# Supplementary material for: TOR–NOT4 signalling couples translation to co-translational quality control in plants
Source: Nat Commun. 2026 Aug 24;17:10101. doi: 10.1038/s41467-026-77200-0 (PMC13598129; doi:10.1038/s41467-026-77200-0)
Supplement: Supplementary file 3 [file 41467_2026_77200_MOESM5_ESM.pdf]

nature portfolio

Corresponding author(s): Daniel J. Gibbs

Last updated by author(s): Jul 16, 2026

Reporting Summary

Nature Portfolio wishes to improve the reproducibility of the work that we publish. This form provides structure for consistency and transparency in reporting. For further information on Nature Portfolio policies, see our Editorial Policies and the Editorial Policy Checklist.

Statistics

For all statistical analyses, confirm that the following items are present in the figure legend, table legend, main text, or Methods section.

n/a	Confirmed	
☐	☒	The exact sample size (n) for each experimental group/condition, given as a discrete number and unit of measurement
☐	☒	A statement on whether measurements were taken from distinct samples or whether the same sample was measured repeatedly
☐	☒	The statistical test(s) used AND whether they are one- or two-sided<br>*Only common tests should be described solely by name; describe more complex techniques in the Methods section.*
☐	☒	A description of all covariates tested
☐	☒	A description of any assumptions or corrections, such as tests of normality and adjustment for multiple comparisons
☐	☒	A full description of the statistical parameters including central tendency (e.g. means) or other basic estimates (e.g. regression coefficient) AND variation (e.g. standard deviation) or associated estimates of uncertainty (e.g. confidence intervals)
☐	☒	For null hypothesis testing, the test statistic (e.g. F, t, r) with confidence intervals, effect sizes, degrees of freedom and P value noted<br>*Give P values as exact values whenever suitable.*
☒	☐	For Bayesian analysis, information on the choice of priors and Markov chain Monte Carlo settings
☒	☐	For hierarchical and complex designs, identification of the appropriate level for tests and full reporting of outcomes
☒	☐	Estimates of effect sizes (e.g. Cohen's d, Pearson's r), indicating how they were calculated

Our web collection on statistics for biologists contains articles on many of the points above.

Software and code

Policy information about availability of computer code

Data collection	Data analysis and visualisation were performed using standard, publicly available software. RNA-seq analyses used HISAT2 v2.0.5, StringTie v1.3.3b, FeatureCounts v1.5.0-p3, DESeq2 v1.20.0, topGO and clusterProfiler, as described in the Methods. Gene Ontology enrichment and graphical visualisation additionally used ShinyGO v0.85.1, VolcaNoseR, Chiplot and Venny 2.0. Protein structure predictions were generated using AlphaFold2, with PyMOL used for visualisation. Image quantification was performed using ImageJ.
Data analysis	*Provide a description of all commercial, open source and custom code used to analyse the data in this study, specifying the version used OR state that no software was used.*

For manuscripts utilizing custom algorithms or software that are central to the research but not yet described in published literature, software must be made available to editors and reviewers. We strongly encourage code deposition in a community repository (e.g. GitHub). See the Nature Portfolio guidelines for submitting code & software for further information.

Data

Policy information about availability of data

All manuscripts must include a data availability statement. This statement should provide the following information, where applicable:

- Accession codes, unique identifiers, or web links for publicly available datasets
- A description of any restrictions on data availability
- For clinical datasets or third party data, please ensure that the statement adheres to our policy

Raw RNA-seq data have been deposited in the Gene Expression Omnibus (GEO) under accession number GSE319908. Complete differential expression tables are provided in the Supplementary Information. Source data underlying all graphs and charts, together with uncropped immunoblots and gels, are provided with this paper. All other data supporting the findings of this study are available from the corresponding author upon reasonable request.

Research involving human participants, their data, or biological material

Policy information about studies with human participants or human data. See also policy information about sex, gender (identity/presentation), and sexual orientation and race, ethnicity and racism.

Reporting on sex and gender	*Use the terms sex (biological attribute) and gender (shaped by social and cultural circumstances) carefully in order to avoid confusing both terms. Indicate if findings apply to only one sex or gender; describe whether sex and gender were considered in study design; whether sex and/or gender was determined based on self-reporting or assigned and methods used. Provide in the source data disaggregated sex and gender data, where this information has been collected, and if consent has been obtained for sharing of individual-level data; provide overall numbers in this Reporting Summary. Please state if this information has not been collected. Report sex- and gender-based analyses where performed, justify reasons for lack of sex- and gender-based analysis.*
Reporting on race, ethnicity, or other socially relevant groupings	*Please specify the socially constructed or socially relevant categorization variable(s) used in your manuscript and explain why they were used. Please note that such variables should not be used as proxies for other socially constructed/relevant variables (for example, race or ethnicity should not be used as a proxy for socioeconomic status). Provide clear definitions of the relevant terms used, how they were provided (by the participants/respondents, the researchers, or third parties), and the method(s) used to classify people into the different categories (e.g. self-report, census or administrative data, social media data, etc.) Please provide details about how you controlled for confounding variables in your analyses.*
Population characteristics	*Describe the covariate-relevant population characteristics of the human research participants (e.g. age, genotypic information, past and current diagnosis and treatment categories). If you filled out the behavioural & social sciences study design questions and have nothing to add here, write "See above."*
Recruitment	*Describe how participants were recruited. Outline any potential self-selection bias or other biases that may be present and how these are likely to impact results.*
Ethics oversight	*Identify the organization(s) that approved the study protocol.*

Note that full information on the approval of the study protocol must also be provided in the manuscript.

Field-specific reporting

Please select the one below that is the best fit for your research. If you are not sure, read the appropriate sections before making your selection.

☒ Life sciences ☐ Behavioural & social sciences ☐ Ecological, evolutionary & environmental sciences

For a reference copy of the document with all sections, see nature.com/documents/nr-reporting-summary-flat.pdf

Life sciences study design

All studies must disclose on these points even when the disclosure is negative.

Sample size	Sample sizes were based on previous experience with comparable Arabidopsis physiological, molecular and biochemical assays routinely performed in our laboratories, and are consistent with those commonly reported in the plant biology literature. No formal statistical method was used to predetermine sample size. The number of biological replicates and sample sizes for each experiment are indicated in the corresponding figure legends. For phenotypic analyses, multiple independent plants or seedlings were analysed for each genotype and treatment. RNA-seq experiments were performed using three independent biological replicates per condition.
Data exclusions	No data were excluded from the analyses unless technical failure prevented collection of interpretable data (for example, unsuccessful immunoblots). No experimental data meeting quality-control criteria were omitted from statistical analyses.
Replication	All key findings were independently replicated. Molecular and biochemical experiments (including immunoblotting, qRT-PCR, proteasome activity assays and puromycin incorporation assays) were performed using independent biological replicates, as indicated in the figure legends. RNA-seq analyses were performed using three independent biological replicates per condition. Phenotypic assays were repeated

independently and yielded consistent results. All attempts at replication were successful.

Randomization	Plants or seedlings were assigned to experimental treatments after germination and grown under identical controlled environmental conditions. For comparative experiments, different genotypes and treatments were grown simultaneously under the same conditions to minimise environmental variation. Random allocation of samples was not required because genotype constituted the experimental variable, but identical handling and growth conditions were maintained throughout each experiment.
Blinding	Blinding was not performed because the experimental variable was plant genotype, which was readily identifiable throughout the study. Quantitative measurements (for example root length, western blot quantification, qRT-PCR and RNA-seq analyses) were based on objective measurements rather than subjective scoring.

Reporting for specific materials, systems and methods

We require information from authors about some types of materials, experimental systems and methods used in many studies. Here, indicate whether each material, system or method listed is relevant to your study. If you are not sure if a list item applies to your research, read the appropriate section before selecting a response.

Materials & experimental systems

n/a	Involved in the study	
☐	☒	Antibodies
☒	☐	Eukaryotic cell lines
☒	☐	Palaeontology and archaeology
☒	☐	Animals and other organisms
☒	☐	Clinical data
☒	☐	Dual use research of concern
☐	☒	Plants

Methods

n/a	Involved in the study	
☒	☐	ChIP-seq
☒	☐	Flow cytometry
☒	☐	MRI-based neuroimaging

Antibodies

Antibodies used	The following primary antibodies were used in this study: anti-RPS6 (Agrisera, AS19 4292), anti-phospho-RPS6 (Agrisera, AS19 4291), anti-UBCJ2 (Enzo Life Sciences, BML-PW8810-0500), anti-GFP/YFP (Roche, 11814460001), anti-HA (Sigma-Aldrich, H3663), anti-ubiquitin (Agrisera, AS08 307A), anti-RPN10 (Agrisera, AS19 4266), anti-RPT4a (Agrisera, AS19 4263), and anti-puromycin (Merck, MABE343). Horseradish peroxidase-conjugated anti-rabbit (Cell Signaling Technology, 7074) and anti-mouse (Sigma-Aldrich, A5278) antibodies were used as secondary antibodies. Antibody dilutions are provided in the Methods.
Validation	Commercial antibodies were used according to the manufacturers' recommendations or published applications. Antibodies recognising Arabidopsis proteins (including RPS6, phospho-RPS6, RPN10 and RPT4a) have been widely used in previous plant studies. Specificity of immunoblot signals was assessed by the expected molecular weight of the detected proteins, appropriate loading controls (Coomassie Brilliant Blue, Rubisco or Stain-Free imaging as indicated), and reproducibility across independent biological replicates. Quantification was performed only on bands corresponding to the desired protein(s).

Dual use research of concern

Policy information about dual use research of concern

Hazards

Could the accidental, deliberate or reckless misuse of agents or technologies generated in the work, or the application of information presented in the manuscript, pose a threat to:

No	Yes	
☒	☐	Public health
☒	☐	National security
☒	☐	Crops and/or livestock
☒	☐	Ecosystems
☒	☐	Any other significant area

Experiments of concern

Does the work involve any of these experiments of concern:

No	Yes	
☒	☐	Demonstrate how to render a vaccine ineffective
☒	☐	Confer resistance to therapeutically useful antibiotics or antiviral agents
☒	☐	Enhance the virulence of a pathogen or render a nonpathogen virulent
☒	☐	Increase transmissibility of a pathogen
☒	☐	Alter the host range of a pathogen
☒	☐	Enable evasion of diagnostic/detection modalities
☒	☐	Enable the weaponization of a biological agent or toxin
☒	☐	Any other potentially harmful combination of experiments and agents

Plants

Seed stocks

The Arabidopsis thaliana lines used in this study were Columbia-0 (Col-0) wild type together with previously described prt6-1 and dcp5 mutants. The not4a (GABI_134E03; GABI-KAT collection), not4b (SAIL_274_D03; SAIL collection) and not4c (SALK_093123; SALK collection) T-DNA insertion lines were obtained from the Nottingham Arabidopsis Stock Centre (NASC). All seed stocks were maintained under controlled growth conditions as described in the Methods.

Novel plant genotypes

Double and triple not4 mutant combinations were generated by genetic crossing of the validated single mutant lines. Transgenic lines expressing pNOT4::NOT4-GUS reporter constructs were generated by Gateway cloning followed by Agrobacterium-mediated floral dip transformation. For complementation of not4a, a 35S::NOT4A-eGFP construct was introduced by Agrobacterium-mediated floral dip. Independent transformants were advanced to the T3 generation, and homozygous lines were identified prior to phenotypic analysis.

Authentication

Homozygous T-DNA insertions were confirmed by PCR genotyping using gene-specific and T-DNA border primers. Loss of full-length NOT4 transcripts was independently confirmed by RT-qPCR and RNA-seq. Following identification by others of an unrelated deletion affecting the PGR3 locus in previously isolated material derived from the GABI_134E03 not4a line, the not4a allele was independently re-isolated from the original NASC seed stock. Following backcrossing to Col-0, homozygous not4a plants were reselected and the absence of additional deletions at the PGR3 locus or neighbouring genomic regions was confirmed by PCR-based genotyping and RNA-seq. The developmental phenotype of not4a was further validated by RNA-seq and complementation with a 35S::NOT4A-eGFP transgene, which substantially rescued the primary root phenotype.
