# Supplementary material for: TOR–NOT4 signalling couples translation to co-translational quality control in plants
Source: Nat Commun. 2026 Aug 24;17:10101. doi: 10.1038/s41467-026-77200-0 (PMC13598129; doi:10.1038/s41467-026-77200-0)
Supplement: Supplementary file 4 [file 41467_2026_77200_MOESM6_ESM.pdf]

nature portfolio

Peer Review File

TOR–NOT4 signalling couples translation to co-translational quality control in plants

Corresponding Author: Professor Daniel Gibbs

This file contains all reviewer reports in order by version, followed by all author rebuttals in order by version.

Version 0:

Reviewer comments:

Reviewer #1

(Remarks to the Author)
This manuscript identifies three Arabidopsis NOT4 E3 ligases linking co-translational quality control to TOR signaling. not4 mutants show increased TOR activity, polyubiquitylated protein accumulation, hypersensitivity to TOR inhibition, and enhanced resistance to Pseudomonas syringae. While the study is interesting and well written, the conclusions lack support from several critical experiments.

1.In Line 26, the authors claim that their findings uncover a previously uncharacterized TOR–NOT4 signaling axis. A critical requirement for establishing this axis is demonstrating how TOR regulates NOT4. Although putative phosphorylation sites were predicted, direct experimental evidence supporting this regulation is lacking.
2.Figure 1: The authors identified three NOT4 homologs in Arabidopsis and examined their growth phenotypes using single, double, and triple combination mutants. However, accumulating evidence indicates that T-DNA insertion mutants may harbor additional mutations or may not solely represent loss-of-function of the targeted gene. For instance, a recent study reported that some previously attributed functions of the well-characterized BIK1 may be inaccurate due to potential genomic perturbations in bik1 T-DNA insertion mutant lines (Song, J. et al. 2026, New alleles of Arabidopsis BIK1 reinforce its predominant role in pattern-triggered immunity and caution interpretations of other reported functions, Nature Plants 12, 284-293). Therefore, it is necessary to test whether NOT4 can complement the observed mutant phenotypes or to use an alternative allele to validate the findings.

3.To test whether NOT4 function is required for plant responses under biologically relevant conditions, the authors examined disease resistance in not4 mutants against Pst. This analysis was largely motivated by a reference (González-Fuente et al., 2025, bioRxiv), in which dcp5 mutants, lacking DCP5, a component of the mRNA decapping complex that functions within processing bodies (P-bodies) to repress translation, exhibit enhanced resistance to Pst. The authors found that, similar to dcp5, not4 mutants were more resistant to Pst infection (Figure 6). Given that loss of NOT4 function increases TOR activity and translation rate (Figure 5), the authors conclude that“increased translation rates in not4abc provide resistance to Pst infection.” However, numerous studies have shown that enhanced TOR activity is negatively correlated with defense; pharmacological or genetic inhibition of TOR activity enhances plant resistance (see references below). Therefore, increased protein synthesis rates in not4 mutants may not be the direct cause of protection against bacterial pathogens.
Aznar et al., TOR signaling downregulation increases resistance to the cereal killer Fusarium graminearum. Plant Signal Behav 2018, 13:e1414120.
De Vleesschauwer et al., Target of rapamycin signaling orchestrates growth-defense trade-offs in plants. New Phytol 2018, 217:305-319.
Chen et al., Cucumber (Cucumis sativus L.) translationally controlled tumor protein interacts with CsRab11A and promotes activation of target of rapamycin in response to Podosphaera xanthii. Plant J 2024, 119:332-347.
Li et al., Posttranslational regulation of TOR kinase activity controls resource allocation between plant growth and immunity in Arabidopsis. Mol Plant 2026, 19:629-650.
4.Line 232: “despite elevated basal TOR activity, not4 mutants are paradoxically hypersensitive to direct TOR inhibition, revealing a decoupling between TOR activation and the effective execution of TOR-dependent outputs.” The conclusion is overly speculative.

Reviewer #2

(Remarks to the Author)
This study identifies three NOT4-like E3 ubiquitin ligases in Arabidopsis thaliana as crucial regulators of co-translational protein quality control. While NOT4 ligases have been studied in other eukaryotic organisms, little remains known about their function in plants. The authors report here a functional link between NOT4 and TARGET OF RAPAMYCIN (TOR) signalling, which coordinates translational output with protein quality control to prevent proteotoxic stress. Loss of NOT4 function results in increased basal TOR activity and accelerated translation rates, causing an accumulation of polyubiquitylated proteins and heightened sensitivity to proteasome and TOR inhibition. They conclude that NOT4 proteins are necessary for normal TOR-dependent signalling. Indeed, mutant not4 plants display similar transcriptomes as TOR-inhibited wild-type plants, further suggesting an important role of NOT4 ligases in balancing translational demands and maintaining proteostasis. Lastly the authors assess the response of not4 mutants to a 'real-world' stress in the form of pathogen infection. They show that elevated translation in NOT4-deficient plants provides resistance to the plant pathogen Pseudomonas syringae, which is known to target host translation. The manuscript culminates into an attractive model that proposes that NOT4 function is in tune with TOR activity to ensure co-translational quality control is scaled in relation to translational demand and output. Thus, the manuscript presents an excellent and comprehensive characterisation of NOT4 function in plants, demonstrating both plant-specific and potential evolutionary conserved mechanisms. I have only a few points for consideration:

1. Fig 1c: The sizes of the HA-tagged AtNOTs are not as expected. This may well be because of an unknown modification in yeast or even auto-monoubiquitination but no mention is currently made of this discrepancy in the text.
Moreover, AtNOT4c does not impact ScCOG1-GFP levels. Is there something to be learned here (in terms of substrate selection) from the amino acid differences between the NOT4 variants?

2. Fig 3e: This figure is not completely clear, which is largely due to the Rubisco loading control changing too much during the assay. A different, more stable loading control would need to be used (e.g. tubulin or actin) to make accurate conclusions on the responses of Rpn10 and Rpt4 in response to proteasome inhibition.

3. The paradox of elevated TOR activity but hypersensitivity to TOR inhibition in not4abc mutants is still somewhat puzzling, although I do accept the authors' conclusion that NOTs influence TOR-dependent signalling outputs. However, given that different NOT4 isoforms have specific subfunctions, I think it is worthwhile assessing the sensitivities of single mutants to proteotoxic stressors (especially isoform b versus a&c). This may allow the authors to better couple specific NOT4 isoforms to TOR-mediated QC-related functions.

4. Given AtNOT4's ability to complement yeast, can the authors speculate in their discussion on potential substrates of the NOT4 ligase? Are homologous substrates present in Arabidopsis?

Reviewer #3

(Remarks to the Author)
This is a very interesting study on the identification of genes and genetic interactions that regulate proteostasis through an interplay between translational levels and protein quality control. The effects of Genetic disruption of these interactions has negative effects on plant growth, but, perhaps unexpectedly, enhances plant resistance to a bacterial pathogen.

In general, the experimental work is of high quality and the results support the conclusions and the proposed picture of interactions and signalling pathways. However, I present below some questions, which should be answered to give a clearer interpretation of the experimental results and to avoid misinterpretations.

1. Lines 125-127. The authors should be more precise in their statement. Figure 1g indicates that not all mutants have statistically significantly reduced primary root length. For example, this is evident for not4bc, which also difficult to distinguish from WT in Fig. S2a.
2. The CHX part of the experiment in Fig 2e is not mentioned in the text and is not discussed.
3. The calculations in Fig 3h should be made differently: for each genotype, the root length should be expressed as a percentage of the corresponding untreated sample, not as an absolute length.
4. Lines 192-194 and Fig. 3i,j. In untreated samples, BiP1/2 and CNX seems actually underexpressed in not4abc with respect to Col-0. Any comment and hypothesis on this?
5. Line 194. Fig. S3h is not related to tunicamycin treatment.
6. Lines 225-238. Fig.S5 is incorrectly mentioned as Fig. S4 in all these lines.
7. Fig. 6 indicates that not4abc is more resistant to infection in spite of the fact that infection decreases protein synthesis to comparable levels in both Col-0 and not4abc. This needs some comments.

Version 1:

Reviewer comments:

Reviewer #1

(Remarks to the Author)
The authors have addressed all my comments.

Reviewer #2

(Remarks to the Author)
In response to my comments, the authors have delivered excellent explanations for their approaches, modified or moderated the manuscript text in key places, and performed additional convincing experimentation to provide further proof for their conclusions. The manuscript has significantly improved and provides exciting new insights into how protein quality control is scaled with translational demand to regulate proteome homeostasis in plants. I have no further questions or comments.

Reviewer #3

(Remarks to the Author)
The concerns I had expressed about the previous version of this manuscript have been thoroughly addressed in the new version. The resulting revised text and figures are now satisfactory from my point of view.

Open Access This Peer Review File is licensed under a Creative Commons Attribution 4.0 International License, which permits use, sharing, adaptation, distribution and reproduction in any medium or format, as long as you give appropriate credit to the original author(s) and the source, provide a link to the Creative Commons license, and indicate if changes were made.
In cases where reviewers are anonymous, credit should be given to 'Anonymous Referee' and the source.
The images or other third party material in this Peer Review File are included in the article's Creative Commons license, unless indicated otherwise in a credit line to the material. If material is not included in the article's Creative Commons license and your intended use is not permitted by statutory regulation or exceeds the permitted use, you will need to obtain permission directly from the copyright holder.
To view a copy of this license, visit https://creativecommons.org/licenses/by/4.0/

Schwarze et al., Response to Reviewers

We thank the editor and all three reviewers for their careful evaluation of our manuscript and for their highly constructive and thoughtful comments. We are particularly encouraged by the reviewers' positive assessment of the significance, quality, and broad interest of the work, including recognition of the study as an important contribution to understanding the integration of translational, co-translational quality control and TOR signalling in plants. We appreciate the reviewers' suggestions, which we feel have helped us to strengthen and clarify the manuscript further.

In response to the reviewers' comments, we have revised the manuscript. These revisions include clarification and moderation of several mechanistic statements, additional data, improvements to figure presentation and quantification, and expanded discussion of the broader implications and limitations of the study. We believe these changes have substantially strengthened the manuscript and addressed the reviewers' concerns.

Below, we provide a detailed point-by-point response to each comment. Reviewer comments are reproduced in black text, followed by our responses in blue.

REVIEWER COMMENTS

Reviewer #1 (Remarks to the Author):

This manuscript identifies three Arabidopsis NOT4 E3 ligases linking co-translational quality control to TOR signaling. not4 mutants show increased TOR activity, polyubiquitylated protein accumulation, hypersensitivity to TOR inhibition, and enhanced resistance to Pseudomonas syringae. While the study is interesting and well written, the conclusions lack support from several critical experiments.

1.In Line 26, the authors claim that their findings uncover a previously uncharacterized TOR–NOT4 signaling axis. A critical requirement for establishing this axis is demonstrating how TOR regulates NOT4. Although putative phosphorylation sites were predicted, direct experimental evidence supporting this regulation is lacking.

We thank the reviewer for this comment and agree that defining the precise molecular mechanism by which TOR regulates NOT4 is an important future direction.

We would first like to clarify that the phosphorylation sites discussed in the manuscript and highlighted in Fig. S4 are not putative computational predictions, but experimentally identified phosphorylation events reported in published phosphoproteomic datasets and curated in PTM Viewer. In particular, phosphorylation of NOT4A at Ser886 was previously shown to be quantitatively TOR-dependent following AZD8055 and rapamycin treatment (Van Leene et al., 2019), while NOT4B was identified as a physical interactor of the TORC1 component LST8 in the same work.

The key advance of this study is the identification of a functional connection between TOR signalling, NOT4 activity, and co-translational QC in plants. Directly establishing which phosphorylation sites across the three NOT4 paralogs are TOR-dependent, generating corresponding phosphosite mutants, and determining their functional significance would constitute a substantial mechanistic study in its own right and falls beyond the scope of the present work. We have therefore revised the manuscript to more clearly distinguish the functional relationship identified here from the underlying biochemical mechanism, which remains to be resolved. Specifically, we now refer more consistently to a functional

connection between TOR and NOT4 that is supported by prior phosphoproteomic evidence and the genetic, biochemical and transcriptomic data presented in this study, while avoiding any implication that the direct biochemical mechanism linking TOR and NOT4 has been established. Accordingly, we have softened the wording in the Abstract and made minor revisions throughout the Discussion to better reflect the scope of our conclusions.

2.Figure 1: The authors identified three NOT4 homologs in Arabidopsis and examined their growth phenotypes using single, double, and triple combination mutants. However, accumulating evidence indicates that T-DNA insertion mutants may harbor additional mutations or may not solely represent loss-of-function of the targeted gene. For instance, a recent study reported that some previously attributed functions of the well-characterized BIK1 may be inaccurate due to potential genomic perturbations in bik1 T-DNA insertion mutant lines (Song, J. et al. 2026, New alleles of Arabidopsis BIK1 reinforce its predominant role in pattern-triggered immunity and caution interpretations of other reported functions, Nature Plants 12, 284-293). Therefore, it is necessary to test whether NOT4 can complement the observed mutant phenotypes or to use an alternative allele to validate the findings.

We thank the reviewer for raising this point. We agree that careful validation of T-DNA insertion mutants is important, particularly in light of recent studies such as the example highlighted by the reviewer. To directly address this concern, we introduced a *35S::NOT4A-eGFP* transgene into the *not4a* mutant background. This substantially rescued the reduced primary root phenotype of *not4a*, consistent with the observed developmental phenotype arising from disruption of NOT4A. We have now added these data as new Fig. S2a,b:

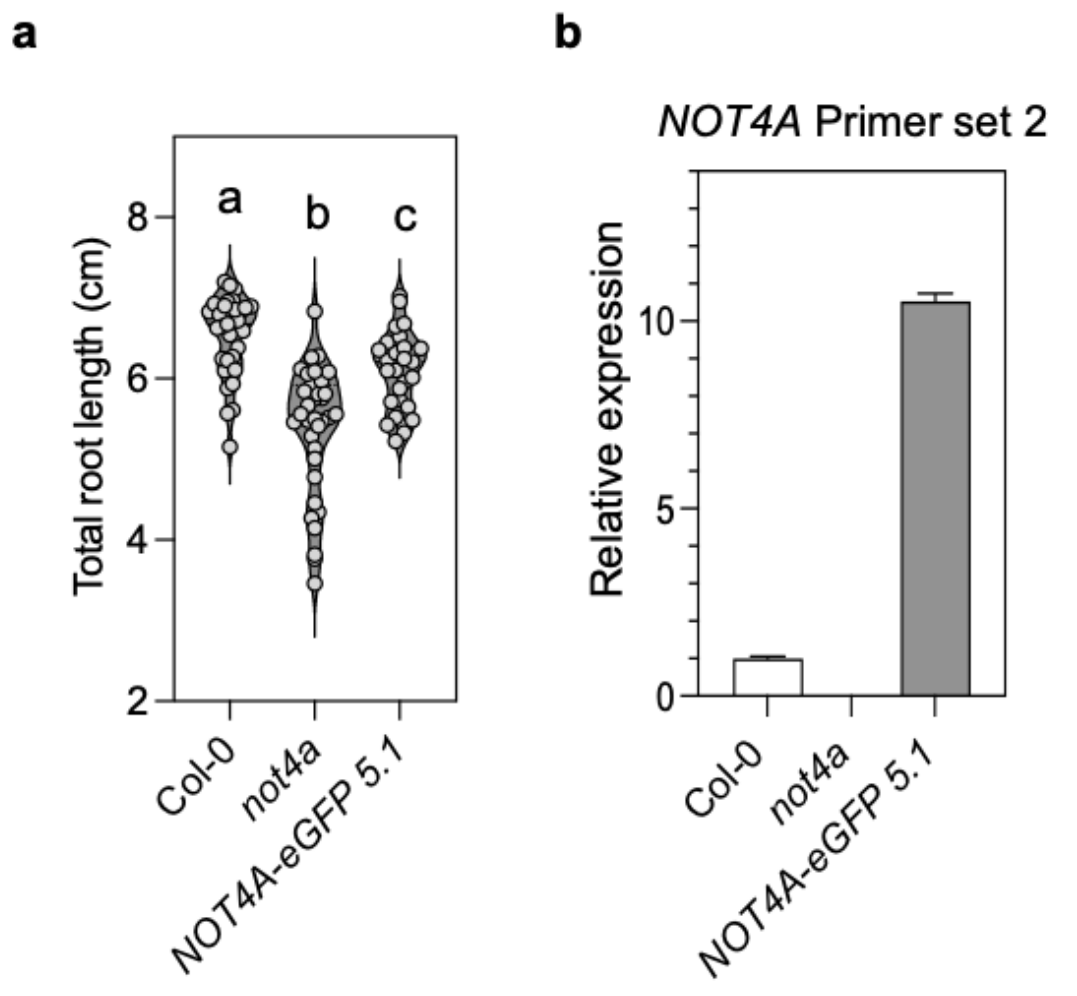

Figure S2: *(**a**) Primary root length of 7-day-old Col-0, not4a, and 35S::NOT4A-eGFP/not4a seedlings grown on ½ MS medium. Different letters indicate statistically significant differences determined by one-way ANOVA followed by Tukey's multiple comparison test (P < 0.05). (**b**) RT-qPCR analysis confirming expression of NOT4A in the 35S::NOT4A-eGFP/not4a complementation line. Expression was normalised to ACTIN2 and is shown relative to Col-0. Data represent mean ± s.d. from n = 3 biological replicates.*

Line 126: *"To further validate that the observed developmental phenotype results from disruption of NOT4A, we introduced a 35S::NOT4A-eGFP transgene into the not4a background and observed substantial rescue of the reduced primary root phenotype (Fig. S2a and b)."*

In addition to this complementation experiment, several independent lines of evidence support the conclusion that the phenotypes described in this study are attributable to disruption of NOT4 function rather than unrelated background mutations. First, we confirmed loss of the corresponding full-length transcripts in the mutant lines by RT-PCR/RT-qPCR and RNA-seq. Second, the developmental, biochemical, molecular and transcriptomic phenotypes are highly internally consistent, including elevated TOR activity, increased translation rates, accumulation of polyubiquitylated proteins, hypersensitivity to proteotoxic stress, and extensive transcriptional reprogramming. Finally, these observations closely parallel established functions of NOT4 proteins in yeast and metazoans, including roles in translational regulation, co-translational quality control and proteostasis, providing further independent support for the biological interpretation. Furthermore, the consistent observation of related phenotypes across multiple independently generated mutant combinations provides additional evidence that these effects arise from disruption of NOT4 function rather than unrelated background mutations.

Although the strongest molecular phenotypes arise in the partially redundant higher-order mutant combinations, comprehensive complementation of these genotypes would require simultaneous reintroduction of multiple NOT4 paralogs. We therefore believe that the new complementation experiment, together with the multiple independent genetic, molecular, biochemical and phenotypic observations presented throughout the manuscript, provides strong support that the reported phenotypes arise from disruption of NOT4 function.

3.To test whether NOT4 function is required for plant responses under biologically relevant conditions, the authors examined disease resistance in not4 mutants against Pst. This analysis was largely motivated by a reference (González-Fuente et al., 2025, bioRxiv), in which dcp5 mutants, lacking DCP5, a component of the mRNA decapping complex that functions within processing bodies (P-bodies) to repress translation, exhibit enhanced resistance to Pst. The authors found that, similar to dcp5, not4 mutants were more resistant to Pst infection (Figure 6). Given that loss of NOT4 function increases TOR activity and translation rate (Figure 5), the authors conclude that“increased translation rates in not4abc provide resistance to Pst infection.” However, numerous studies have shown that enhanced TOR activity is negatively correlated with defense; pharmacological or genetic inhibition of TOR activity enhances plant resistance (see references below). Therefore, increased protein synthesis rates in not4 mutants may not be the direct cause of protection against bacterial pathogens.

Aznar et al., TOR signaling downregulation increases resistance to the cereal killer Fusarium graminearum. Plant Signal Behav 2018, 13:e1414120.
De Vleesschauwer et al., Target of rapamycin signaling orchestrates growth-defense trade-offs in plants. New Phytol 2018, 217:305-319.
Chen et al., Cucumber (Cucumis sativus L.) translationally controlled tumor protein interacts with CsRab11A and promotes activation of target of rapamycin in response to Podosphaera xanthii. Plant J 2024, 119:332-347.
Li et al., Posttranslational regulation of TOR kinase activity controls resource allocation between plant growth and immunity in Arabidopsis. Mol Plant 2026, 19:629-650.

We thank the reviewer for this comment and agree that TOR signalling has complex and context-dependent roles in balancing growth and immunity. Our interpretation is not that elevated TOR activity universally promotes disease resistance, but rather that altered translational homeostasis in *not4abc* may contribute to resistance in the specific context of a pathogen that suppresses host translation. This interpretation is supported by the elevated basal translational activity observed in *not4abc* together with the finding that inhibition of translation by cycloheximide abolishes the enhanced resistance phenotype. To further clarify this point, we have softened the corresponding statements in the manuscript.

Line 304: Thus, elevated basal translational activity in the absence of NOT4 function may contribute to protection against a bacterial pathogen that suppresses host translation.

We have also expanded the Discussion to acknowledge the broader and context-dependent relationship between TOR signalling and plant immunity and added the reviewer-suggested references.

Line 423: More broadly, TOR signalling has complex and context-dependent roles in plant immunity, with both positive and negative effects on disease resistance reported depending on the host–pathogen interaction and physiological context[58-61].

4.Line 232: "despite elevated basal TOR activity, not4 mutants are paradoxically hypersensitive to direct TOR inhibition, revealing a decoupling between TOR activation and the effective execution of TOR-dependent outputs." The conclusion is overly speculative.

We thank the reviewer for this comment. To further clarify the interpretation of these findings, we have modified this statement in the revised manuscript to indicate that the observed relationship between elevated basal TOR activity and hypersensitivity to TOR inhibition is consistent with, rather than definitively demonstrating, a partial uncoupling between TOR activation and downstream TOR-dependent outputs.

Line 244: "*Thus, despite elevated basal TOR activity, not4 mutants were paradoxically hypersensitive to direct TOR inhibition, consistent with a partial uncoupling between TOR activation and the effective execution of TOR-dependent outputs*."

Reviewer #2 (Remarks to the Author):

This study identifies three NOT4-like E3 ubiquitin ligases in Arabidopsis thaliana as crucial regulators of co-translational protein quality control. While NOT4 ligases have been studied in other eukaryotic organisms, little remains known about their function in plants. The authors report here a functional link between NOT4 and TARGET OF RAPAMYCIN (TOR) signalling, which coordinates translational output with protein quality control to prevent proteotoxic stress. Loss of NOT4 function results in increased basal TOR activity and accelerated translation rates, causing an accumulation of polyubiquitylated proteins and heightened sensitivity to proteasome and TOR inhibition. They conclude that NOT4 proteins are necessary for normal TOR-dependent signalling. Indeed, mutant not4 plants display similar transcriptomes as TOR-inhibited wild-type plants, further suggesting an important role of NOT4 ligases in balancing translational demands and maintaining proteostasis. Lastly the authors assess the response of not4 mutants to a 'real-world' stress in the form of pathogen infection. They show that elevated translation in NOT4-deficient plants provides resistance to the plant pathogen Pseudomonas syringae, which is known to target host translation. The manuscript culminates into an attractive model that proposes that NOT4 function is in tune with TOR activity to ensure co-translational quality control is scaled in relation to translational demand and output. Thus, the manuscript presents an excellent and comprehensive characterisation of NOT4 function in plants, demonstrating both plant-specific and potential evolutionary conserved mechanisms. I have only a few points for consideration:

1. Fig 1c: The sizes of the HA-tagged AtNOTs are not as expected. This may well be because of an unknown modification in yeast or even auto-monoubiquitination but no mention is currently made of this discrepancy in the text.
Moreover, AtNOT4c does not impact ScCOG1-GFP levels. Is there something to be learned

here (in terms of substrate selection) from the amino acid differences between the NOT4 variants?

We thank the reviewer for these comments. With respect to the migration of the HA-tagged *At*NOT4 proteins differing from their predicted molecular weights, whilst we have not directly investigated the basis of this mobility shift, it may plausibly reflect post-translational modification, as suggested by the reviewer (e.g., phosphorylation or ubiquitylation). Indeed, in Arabidopsis all three paralogs contain multiple experimentally identified phosphorylation sites (Fig. S4), and these proteins may also undergo post-translational modification in the heterologous yeast system, contributing to altered electrophoretic mobility. Alternatively, anomalous SDS-PAGE migration associated with the extended intrinsically disordered regions present in the Arabidopsis NOT4 proteins may also play a role, as has been described for other disordered proteins (e.g., Schramm *et al*., 2019[1])

We also agree that the inability of AtNOT4C to reduce ScCOG1-GFP levels relative to AtNOT4A/B may point towards functional divergence between the paralogs. One possibility is that sequence variation outside the conserved RING and RRM domains influences substrate recognition or interaction with components of the yeast CCR4–NOT/co-translational QC machinery. For example, we highlight in the Discussion that yeast NOT4 is thought to function primarily as part of the broader CCR4–NOT complex, whereas in other organisms NOT4 proteins may also operate more independently. Alternatively, AtNOT4C may require plant-specific cofactors or regulatory interactions that are not fully recapitulated in the heterologous yeast system. We have now briefly mentioned the MW discrepancy in the legend of Figure 1C and included brief discussion of these possibilities in the Discussion of the revised manuscript.

Line 331: "*The inability of AtNOT4C to reduce ScCOG1-GFP abundance relative to AtNOT4A/B may further reflect functional divergence linked to sequence variation outside the conserved RING and RRM domains. We also note that AtNOT4s displayed differing electrophoretic mobilities relative to their predicted molecular weights, possibly due to post-translational modification and/or their extended intrinsically disordered regions*[1]."

2. Fig 3e: This figure is not completely clear, which is largely due to the Rubisco loading control changing too much during the assay. A different, more stable loading control would need to be used (e.g., tubulin or actin) to make accurate conclusions on the responses of Rpn10 and Rpt4 in response to proteasome inhibition.

We thank the reviewer for this comment. We agree that the Rubisco loading control shows some variability across treatments in the representative blot. However, the quantified values shown beneath the blots were calculated by normalizing RPN10 and RPT4 signal intensities to the corresponding Rubisco signal for each lane. Following normalization, both proteasome subunits still showed stronger accumulation in *not4abc* relative to Col-0 following bortezomib treatment, and this trend was observed across multiple experimental repeats. We have clarified this normalization procedure in the figure legend and moderated the corresponding text in the manuscript.

Line 189: "*Consistent with this heightened sensitivity, elevated levels of representative proteasome subunits - including RPN10 and RPT4 – were observed in not4abc relative to Col-0 following bortezomib treatment, based on quantification normalized to the corresponding Rubisco loading signal (**Fig. 3e**).*"

3. The paradox of elevated TOR activity but hypersensitivity to TOR inhibition in not4abc

mutants is still somewhat puzzling, although I do accept the authors' conclusion that NOTs influence TOR-dependent signalling outputs. However, given that different NOT4 isoforms have specific subfunctions, I think it is worthwhile assessing the sensitivities of single mutants to proteotoxic stressors (especially isoform b versus a&c). This may allow the authors to better couple specific NOT4 isoforms to TOR-mediated QC-related functions.

We thank the reviewer for this suggestion. We agree that paralog-specific functions are an interesting possibility. Indeed, our TOR inhibitor assays already indicate isoform-specific behaviour, with *not4a/not4c* showing stronger sensitivity to direct TOR inhibition, whereas *not4b* was most sensitive to inhibition of the S6K branch. We have now extended this analysis by assessing the sensitivity of the individual *not4* mutants to the proteasome inhibitor bortezomib. In contrast to the pronounced hypersensitivity of *not4abc*, none of the single mutants displayed enhanced sensitivity relative to Col-0 (new Fig. S2e), consistent with functional redundancy among the NOT4 paralogs in maintaining proteostasis. These findings suggest that while individual NOT4 proteins may make distinct contributions to TOR signalling outputs, the proteotoxic stress phenotypes reported here become apparent only following combined loss of NOT4 function. We have included these new data in Fig. S2e and have clarified this point in the Results and Discussion of the revised manuscript.

New Fig. S2e:

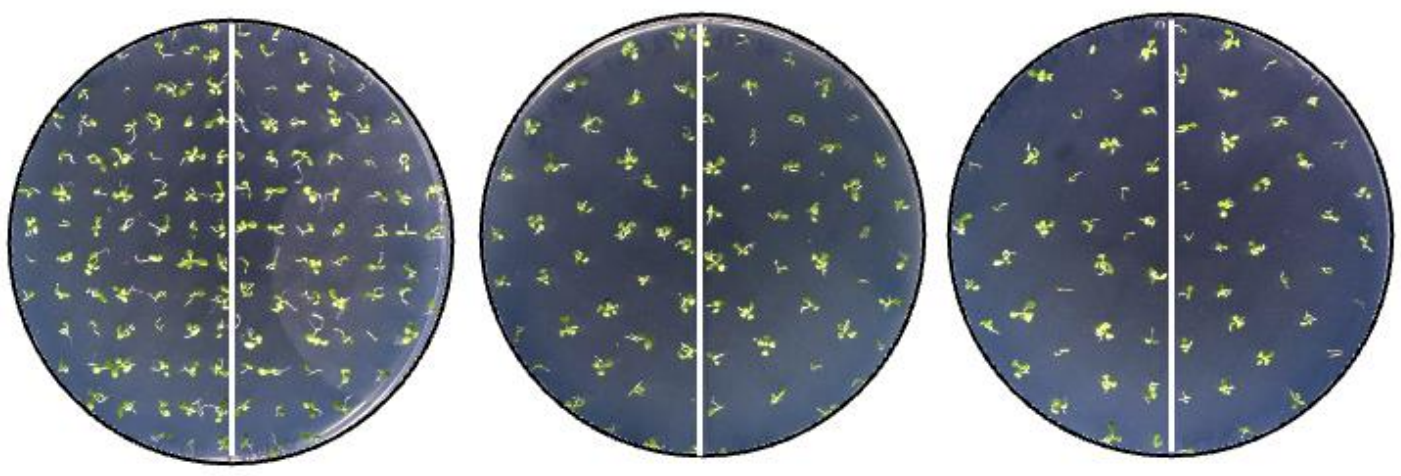

Results, Line 186: *"By contrast, the individual not4 single mutants did not exhibit enhanced sensitivity to bortezomib treatment relative to Col-0 (Fig. S2e), indicating that the proteotoxic stress phenotype only emerges following combined loss of NOT4 function."*

Discussion, Line 350: *"Interestingly, although individual NOT4 paralogs displayed distinct sensitivities to TOR pathway inhibition, none of the corresponding single mutants exhibited enhanced sensitivity to proteasome inhibition (Fig. S2e). This suggests that while individual NOT4 proteins contribute differentially to TOR signalling outputs, maintenance of proteostasis appears to be functionally buffered by redundancy between the NOT4 paralogs, with proteotoxic stress only emerging following combined loss of NOT4 function."*

4. Given AtNOT4's ability to complement yeast, can the authors speculate in their discussion on potential substrates of the NOT4 ligase? Are homologous substrates present in Arabidopsis?

We thank the reviewer for this suggestion. In yeast and animals, NOT4 proteins have been linked to the ubiquitylation and regulation of a broad range of substrates associated with co-translational quality control, proteasome homeostasis, mRNA metabolism and chromatin-associated processes. Given the ability of AtNOT4A/B to partially complement ScNOT4 function in yeast, it is plausible that Arabidopsis NOT4 proteins similarly act on conserved classes of substrates linked to translational surveillance and proteostasis pathways. Indeed, many of the core components of these pathways are themselves conserved in Arabidopsis, although whether they represent direct substrates of plant NOT4 proteins remains unknown.

However, the direct substrates of plant NOT4 proteins remain unknown. We have now expanded the Discussion to highlight this possibility and note that future identification of Arabidopsis NOT4 interacting proteins and ubiquitylation targets will help define the mechanistic conservation and diversification of NOT4 function across eukaryotes.

Line 346: "*Future focus on identifying Arabidopsis NOT4 binding partners and ubiquitylation targets, particularly among factors linked to translational surveillance, proteostasis and proteasome function, will help to further delineate the extent to which plant NOT4 activity is conserved…*"

Reviewer #3 (Remarks to the Author):

This is a very interesting study on the identification of genes and genetic interactions that regulate proteostasis through an interplay between translational levels and protein quality control. The effects of Genetic disruption of these interactions has negative effects on plant growth, but, perhaps unexpectedly, enhances plant resistance to a bacterial pathogen.

In general, the experimental work is of high quality and the results support the conclusions and the proposed picture of interactions and signalling pathways. However, I present below some questions, which should be answered to give a clearer interpretation of the experimental results and to avoid misinterpretations.

1. Lines 125-127. The authors should be more precise in their statement. Figure 1g indicates that not all mutants have statistically significantly reduced primary root length. For example, this is evident for not4bc, which also difficult to distinguish from WT in Fig. S2a.

We thank the reviewer for this comment. We agree that some wording in the manuscript could be interpreted too broadly with respect to the primary root phenotype. While the Results section specifies the mutant combinations exhibiting statistically significant reductions in primary root length (*not4a*, *not4ab*, *not4ac* and *not4abc*; Fig. 1g), we have revised the broader phrasing elsewhere in the manuscript for greater precision.

2. The CHX part of the experiment in Fig 2e is not mentioned in the text and is not discussed.

We thank the reviewer for highlighting this oversight. The cycloheximide (CHX) treatment in Fig. 2e was included as a control to confirm that puromycin incorporation reflects active translation. We have now revised the Results section to explicitly describe the CHX treatment and its effect on puromycin incorporation.

Line 165: "*Following 60 minutes of puromycin treatment, addition of cycloheximide (CHX) suppressed further puromycin incorporation in both genotypes, whereas DMSO-treated seedlings continued to accumulate puromicylated proteins over the subsequent 60 minutes. Together, these observations confirm that the detected signal reflects active translation (**Fig. 2e**).*"

3. The calculations in Fig 3h should be made differently: for each genotype, the root length should be expressed as a percentage of the corresponding untreated sample, not as an absolute length.

We thank the reviewer for this comment and agree that expressing root length relative to the corresponding untreated control is important for comparing genotype-specific sensitivity to tunicamycin treatment. For this reason, we included the normalized relative primary root

length values as the inset graph in Fig. 3h, whilst retaining the absolute root length measurements in the main panel to also illustrate the overall severity of the growth inhibition phenotype. We have now clarified this more explicitly in the figure legend.

4. Lines 192-194 and Fig. 3i,j. In untreated samples, BiP1/2 and CNX seems actually underexpressed in not4abc with respect to Col-0. Any comment and hypothesis on this?

We thank the reviewer for this comment. We agree that *BIP1/2* and *CNX1* show reduced basal expression in untreated not4abc relative to Col-0, and this trend is also consistent with the RNA-seq data for these genes (Fig. S3h). One possible interpretation is that loss of NOT4 function does not constitutively activate the canonical ER UPR under basal conditions, but instead alters proteostasis/translational homeostasis such that not4abc mounts a stronger response when challenged with additional protein-folding stress. Importantly, following tunicamycin treatment, *BIP1/2, CNX1* and *BZIP60* were induced more strongly in *not4abc* relative to Col-0, supporting our conclusion that NOT4-deficient plants are hypersensitive to ER protein-folding stress. We have revised the text to note this observation.

Line 201: "*Basal expression of the canonical UPR marker genes BIP1/2*, *CNX1 and BZIP60 was not elevated in not4abc, with BIP1/2 and CNX1 instead showing modestly reduced expression under untreated conditions, consistent with the RNA-seq data (Fig. S3h). However, all three marker genes were hyperinduced relative to WT in response to tunicamycin treatment (Fig.* ***3i,j****), indicating that not4abc does not constitutively activate the canonical UPR but is hypersensitive to additional ER protein-folding stress.*"

5. Line 194. Fig. S3h is not related to tunicamycin treatment.

We have now corrected this as part of our response to point 4 above.

6. Lines 225-238. Fig.S5 is incorrectly mentioned as Fig. S4 in all these lines.

We thank the reviewer for identifying this error. The figure citations have now been corrected throughout this section of the manuscript.

7. Fig. 6 indicates that not4abc is more resistant to infection in spite of the fact that infection decreases protein synthesis to comparable levels in both Col-0 and not4abc. This needs some comments.

We thank the reviewer for this insightful comment. We agree that Pst infection reduced puromycin incorporation in both Col-0 and *not4abc* to broadly comparable levels at the sampled timepoint. We have now expanded the Discussion to clarify that our interpretation is not that elevated translation is fully maintained throughout infection in *not4abc*, but rather that altered basal translational homeostasis prior to, and during the early stages of infection, may contribute to the enhanced resistance phenotype. We also note that elevated translation is unlikely to be the sole determinant of this phenotype, and that additional NOT4-dependent changes in proteostasis or stress signalling may also contribute to the enhanced resistance observed in n*ot4abc***.**

Line 418: "*Notably, although Pst infection reduced translation rates in both Col-0 and not4abc at the sampled timepoint, altered basal translational homeostasis prior to infection and during early infection stages may nevertheless contribute to the enhanced resistance phenotype observed in not4abc. Additional NOT4-dependent changes in proteostasis or*

stress signalling may also contribute."

References

1. Schramm, A., Bignon, C., Brocca, S., Grandori, R., Santambrogio, C., and Longhi, S. (2019). An arsenal of methods for the experimental characterization of intrinsically disordered proteins - How to choose and combine them? Arch Biochem Biophys *676*, 108055. 10.1016/j.abb.2019.07.020.
